# Explainable AI reveals tissue pathology and psychosocial drivers of opioid prescription for non-specific chronic low back pain

**DOI:** 10.1038/s41598-025-13619-7

**Published:** 2025-08-21

**Authors:** Michelle W. Tong, Katharina Ziegeler, Virginie Kreutzinger, Sharmila Majumdar

**Affiliations:** 1 Department of Radiology and Biomedical Imaging, University of California San Francisco, San Francisco, USA; 2 Department of Bioengineering, University of California Berkeley, Berkeley, CA 94720 USA; 3 Department of Bioengineering and Therapeutic Sciences, University of California San Francisco, San Francisco, USA

**Keywords:** EMR, EHR, Explainable/Interpretable AI, Non-specific chronic lower back pain, Medication, Prescription, Opioid, NSAID, Computational models, Data integration, Data processing, Machine learning, Predictive medicine, Predictive markers, Biomarkers, Health policy, Medical ethics, Quality of life, Medical research, Translational research, Health care, Public health, Epidemiology

## Abstract

Effective management of non-specific chronic lower back pain (ns-cLBP) requires nuanced prescription decisions within evolving guidelines for conservative treatment. This study developed comprehensive LBP patient profiles from electronic medical records (EMR), integrating clinical charts (demographics, social determinants, diagnoses, medications) and radiology reports (MRI-confirmed diagnoses) to predict pharmacological management strategies. One-vs-one and one-vs-rest classification frameworks systematically evaluated treatment decisions across three prescriptions: no medication, NSAIDs, and opioids. Real-world complexity and heterogeneity in ns-cLBP management was reflected in modest yet clinically meaningful performance metrics (balanced accuracy = 0.58, AUC = 0.62, F1-score = 0.42). Chart-documented diagnoses marginally outperformed MRI-reported pathology as predictors, though this difference was within the range of variability, which suggests the importance of diagnoses informed by patient-reported symptoms in shaping treatment pathways. SHAP feature importance analysis identified consistent predictors (*year_at_first_imaging*) and variable factors (*spinal_stenosis*, *disc_pathology*,* race_ethnicity*, *negative_psych_state*, *osteoarthritis_osteoarthrosis*) in prescriptions, with higher associations observed in those with anxiety or depression, partnered individuals and females. By leveraging explainable AI, this study quantifies the interplay between biological and psychosocial drivers of prescribing decisions, offering a transparent, data-driven monitoring tool for understanding in chronic pain care. These findings demonstrate the potential of multi-modal EMR data and interpretable models to guide more personalized, equitable ns-cLBP management and opioid prescriptions.

## Introduction

Lower back pain (LBP) represents a global health crisis, affecting 619 million people worldwide as the leading cause of years lived with disability. This debilitating condition incurs considerable socioeconomic burden due to healthcare costs and individuals’ loss of productivity^1–3^.

The challenge of managing LBP arises in its complex, multifaceted nature. While some cases are attributed to specific pathologies, many persistent cases are classified as non-specific chronic low back pain (ns-cLBP), where traditional diagnostic approaches fail to reveal a single causative and addressable pathology^4,5^. Current clinical practice guidelines advocate for a biopsychosocial approach^6^, acknowledging the complex interplay between patient-reported symptoms, imaging findings, and intervention risks^7,8^ consistent with general pain management^9^. This comprehensive framework relies primarily on physical examination and patient history^5,10^, reserving imaging for cases with red flags or persistent pain beyond 4–6 weeks^5,11^. However, time constraints can limit discussion of thorough patient history and treatment options, and imaging findings have shown weak correlation with LBP^12^. Despite existing research-supported guidelines, their implementation in clinical practice remains inconsistent^13–15^ with imaging often overutilized^5^, highlighting a critical gap between recommended and actual care.

The treatment landscape for LBP presents an even more compelling challenge. Standard conservative approaches often prove insufficient for many patients who experience chronic pain in conjunction with multi-tissue degeneration of the spine^4^, with minimal advancements in treatment options^16^. This therapeutic void has led to increased reliance on pharmacological interventions, particularly non-steroid anti-inflammatory drugs (NSAIDs) and opioids. However, the role of opioids ns-cLBP management has come under intense scrutiny due to limited evidence supporting their long-term efficacy and established concerns about addiction and overdose risks^4,17^. While current guidelines recommend alternative medications as a first line of defense, actual opioid prescription patterns appear to be heavily influenced by individual clinical behavior^18^ and social determinants of health^19,20^, suggesting a more complex decision-making process than previously recognized.

Recent technological advances offer a promising path towards personalized medicine through the assessment of patient characteristics that influence pharmacological management in ns-cLBP. The emergence of multi-modal electronic medical record (EMR) databases and explainable AI^21,22^ have created unprecedented opportunities to analyze real-world ns-cLBP treatment patterns at scale^23^. Modern large language models (LLMs) can now extract nuanced pathology descriptors from clinical notes^24^, while interpretable AI frameworks, like SHapely Additive ePlanations (SHAP)^25^, provide quantitative assessments of factors influencing clinical outcomes^26^. Despite these innovations, no prior study has attempted to predict opioid medication prescription for LBP management, rather most classification studies focus on LBP diagnosis or prognosis^27^. The only related work assessed opioid prescription in the context of opioid use disorder^28^.

This study provides the first comprehensive evaluation on how tissue pathology interacts with social determinants of health in driving medication prescriptions for clinical ns-cLBP patients, through systematic comparison of machine learning models and SHAP-based feature importance.

Our approach integrates:

1. *Comprehensive clinically informed LBP patient profiles* constructed from multi-modal electronic medical records (EMRs), including structured tabular medical chart data and radiology report texts.
2. *Tree-based classification algorithms* trained to predict no, NSAID, and opioid medication prescription using one-vs-one (OVO) and one-vs-rest (OVR) strategies, offering a data-driven perspective on the biopsychosocial factors influencing ns-cLBP intervention. Unlike conventional methods that focus primarily on treatment efficacy, these algorithms reveal medical policies shaping historical prescription patterns.
3. *SHAP-based feature importance assessment* quantified key characteristics associated with medication prescription pathways, offering population-level benchmarks to compare with evidence-based guidelines and to identify underserved target populations for prescribing interventions.

By leveraging advanced natural language processing and machine learning approaches, we aim to uncover associations between patient characteristics and treatment selection behavior. This work presents a transparent, data-driven framework for clinical insights into both pathological and psychosocial drivers of opioid prescription practices, identifies specific EMR documentation improvements needed for AI-assisted decision making, and provides a reference for future initiatives to advance conservative care in ns-cLBP.

## Results

### ns-cLBP cohort and patient profiles

**Fig. 1:**
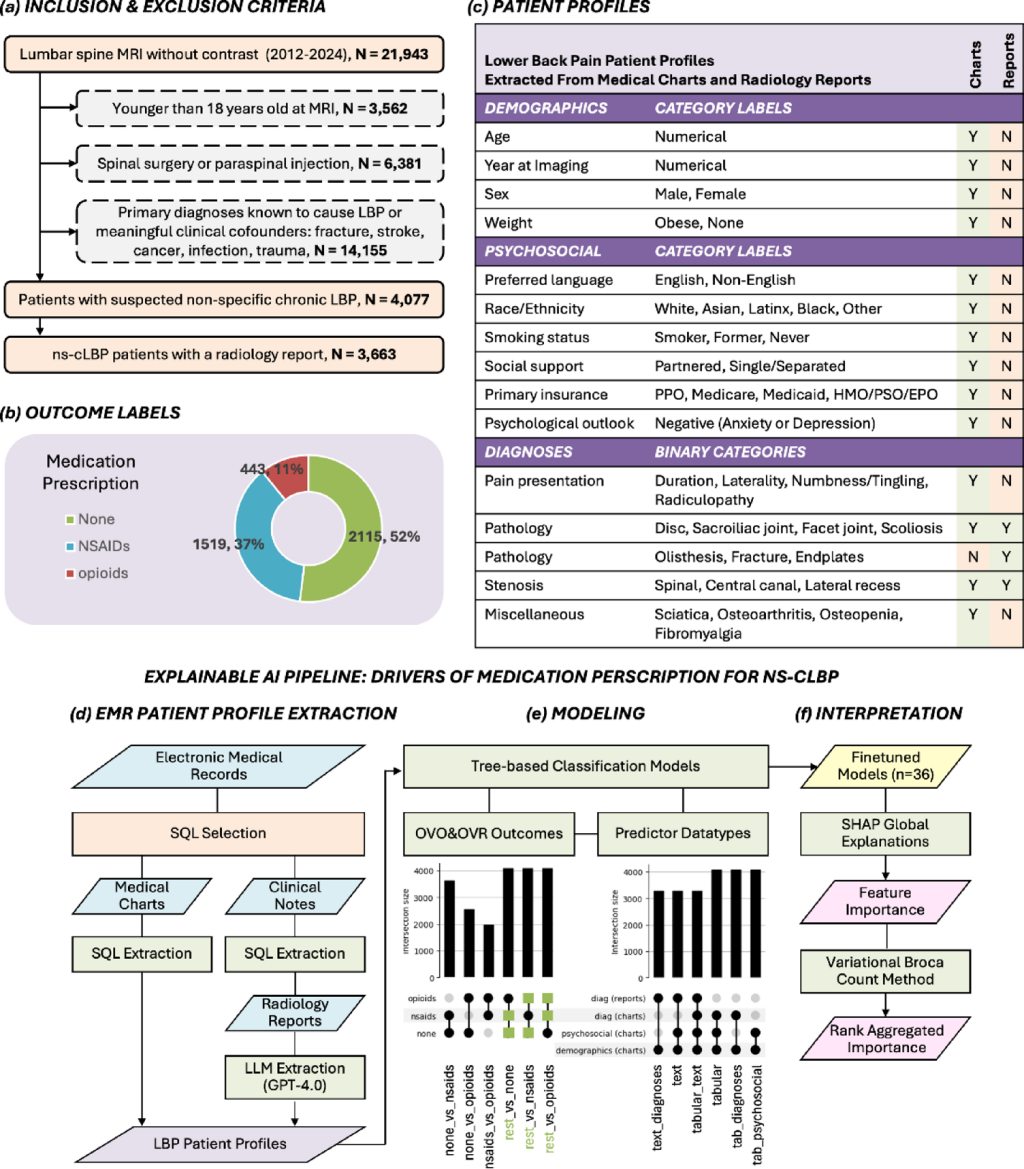
Schematic of Pipeline for EMR Patient Profile Drivers of Medication Prescriptions. (**a**) Cohort selection criteria for suspected non-specific chronic low back pain (ns-cLBP) patients from our institution’s electronic medical records (EMRs). Inclusion (solid orange) and exclusion (dashed gray) criteria were defined with clinical experts to identify patients with lumbar spine MRI between 2012–2024, excluding those with prior interventions, surgery, or underlying conditions requiring specific treatments. Exclusion rational is described in detail in the methods 4.1.1. and exclusion sample sizes (N) are subsets of all patients with lumbar spine MRI. The final cohort included 4,077 ns-cLBP patients, of whom 3,663 had available radiology reports. (**b**) Patients were categorized by the most potent pain medication class (none, NSAID, or opioid) prescribed within one year following their first lumbar spine MRI. (**c**) Patient profiles were constructed from demographic, psychosocial, and diagnostic features up until a month after their first lumbar spine MRI, extracted as numerical, categorical, or binary variables from medical records and radiology reports. Feature selection was guided by clinical expertise and current research recommendations. (**d**) Structured SQL queries were used for tabular data extraction, while large language model (LLM) prompting was applied to text data to construct clinical patient profiles for ns-cLBP. (**e**) Tree-based classifiers were trained for six binary outcome variables and six predictor datatypes specified along the x-axis in the UpSet Diagram. One-vs-one (OVO) comparisons assessed relative medication potency, while one-vs-rest (OVR) comparisons evaluated prescription trends for specific drug classes by comparing each drug class (black circle) to its mathematical complement (green square). Predictor datatypes were defined to assess the influence of biopsychosocial factors and documentation methods with y-axis categories corresponding to patient characteristics in (**c**). Models were optimized based on the highest validation balanced accuracy across model architectures (random forest, bagging, adaboost, and XGBoost) and hyperparameters via grid search. (**f**) SHAP values quantified feature importance, providing insights into clinical predictors of medication prescription. A variational Broca Count method aggregated feature importance across OVO and OVR models to summarize general prescribing patterns beyond pairwise comparisons.

Clinically informed selection criteria identified 4,077 ns-cLBP patients from the EMR of whom 3,286 had a radiology report. Patient prescription distributions aligned with clinical guidelines that favor conservative management spanning no pharmacological intervention (52%), NSAIDs (37%), or opioids (11%) (Fig. 1).

For big-data analysis, a LLM prompt previously developed on radiology reports from our institution extracted the presence of LBP-related pathology from radiology reports. Initial prompt validation on a random sample of 20 reports across 10 pathologies yielded an average 0.956 ± 0.041 F1-score, 0.029 ± 0.035 FPR, and 0.030 ± 0.041 FNR between the LLM extraction pipeline and human annotation (Supplemental Table 1). Such performance metrics demonstrated strong agreement which justified proceeding with processing the entire cohort. Documentation of diagnoses differed between clinical charts and radiology reports with low agreement (Cohen’s kappa = 0.102 ± 0.0183) and more frequent reporting in tabular data where physicians account for patient-reported symptoms (Supplemental Table 2).

Complete patient profile features and statistical distributions of values across medication outcomes are summarized in Table 1.

**Table 1:** Non-specific chronic lower back pain patient profiles.

Feature	Value	ID	*N* (%) or Mean (SD) grouped by intervention				*P*-Value
			Overall (n=3663)	Unspecified (n=1411)	NSAID (n=414)	Opioid (n=1838)	
-- Demographics							
Age at first imaging	**---**	**---**	48.5 (16.6)	49.5 (16.7)	49.8 (15.4)	47.3 (16.8)	< 0.001
Year at first imaging	**---**	**---**	2018.7 (3.0)	2019.1 (2.8)	2018.4 (3.0)	2018.5 (3.2)	< 0.001
Sex	Male	1	2009 (54.8)	744 (52.7)	226 (54.6)	1039 (56.5)	0.097
	Female	0	1654 (45.2)	667 (47.3)	188 (45.4)	799 (43.5)	---
Obesity	Not obese	0	3473 (94.8)	1315 (93.2)	378 (91.3)	1780 (96.8)	< 0.001
	Obese	1	190 (5.2)	96 (6.8)	36 (8.7)	58 (3.2)	---
Psychosocial							
Preferred language	English	1	3433 (93.7)	1312 (93.0)	384 (92.8)	1737 (94.5)	0.144
	Not English	0	230 (6.3)	99 (7.0)	30 (7.2)	101 (5.5)	---
Race / ethnicity	White	1	2093 (57.1)	821 (58.2)	240 (58.0)	1032 (56.1)	< 0.001
	Asian	2	541 (14.8)	242 (17.2)	48 (11.6)	251 (13.7)	---
	Other	0	520 (14.2)	186 (13.2)	48 (11.6)	286 (15.6)	---
	Latinx	3	346 (9.4)	103 (7.3)	56 (13.5)	187 (10.2)	---
	Black	4	163 (4.4)	59 (4.2)	22 (5.3)	82 (4.5)	---
Smoking status	Never	0	2823 (77.1)	1077 (76.3)	293 (70.8)	1453 (79.1)	0.001
	Former	1	643 (17.6)	266 (18.9)	85 (20.5)	292 (15.9)	---
	Smoker	2	197 (5.4)	68 (4.8)	36 (8.7)	93 (5.1)	---
Social support	Partnered	1	2072 (56.6)	844 (59.8)	231 (55.8)	997 (54.2)	0.006
	Single/ separated	0	1591 (43.4)	567 (40.2)	183 (44.2)	841 (45.8)	---
Primary insurance	PPO	4	2008 (54.8)	748 (53.0)	198 (47.8)	1062 (57.8)	< 0.001
	Medicare	2	648 (17.7)	283 (20.1)	72 (17.4)	293 (15.9)	---
	HMP/POS/EPO	3	491 (13.4)	199 (14.1)	62 (15.0)	230 (12.5)	---
	Medicaid	2	438 (12.0)	160 (11.3)	66 (15.9)	212 (11.5)	---
	Other	0	78 (2.1)	21 (1.5)	16 (3.9)	41 (2.2)	---
Negative psych state (anxiety or depression)	Not negative	0	3252 (88.8)	1182 (83.8)	341 (82.4)	1729 (94.1)	< 0.001
	Negative	1	411 (11.2)	229 (16.2)	73 (17.6)	109 (5.9)	**---**
Diagnoses (charts)							
LBP duration	Unspecified	0	2551 (69.6)	869 (61.6)	273 (65.9)	1409 (76.7)	< 0.001
	Chronic	2	896 (24.5)	455 (32.2)	90 (21.7)	351 (19.1)	---
	Acute	1	216 (5.9)	87 (6.2)	51 (12.3)	78 (4.2)	---
LBP laterality	Unspecified	0	2681 (73.2)	912 (64.6)	293 (70.8)	1476 (80.3)	< 0.001
	Unilateral	2	552 (15.1)	281 (19.9)	81 (19.6)	190 (10.3)	---
	Bilateral	1	430 (11.7)	218 (15.5)	40 (9.7)	172 (9.4)	---
Numbness / tingling	Present	1	258 (7.0)	116 (8.2)	24 (5.8)	118 (6.4)	0.08
Radiculopathy	Present	1	1697 (46.3)	753 (53.4)	215 (51.9)	729 (39.7)	< 0.001
Disc pathology	Present	1	1617 (44.1)	659 (46.7)	227 (54.8)	731 (39.8)	< 0.001
Spinal stenosis	Present	1	1080 (29.5)	509 (36.1)	163 (39.4)	408 (22.2)	< 0.001
Facet joint arthropathy	Present	1	306 (8.4)	161 (11.4)	35 (8.5)	110 (6.0)	< 0.001
Sacroiliac joint	Present	1	140 (3.8)	73 (5.2)	9 (2.2)	58 (3.2)	0.002
Scoliosis	Present	1	289 (7.9)	120 (8.5)	37 (8.9)	132 (7.2)	0.269
Sciatica	Present	1	1089 (29.7)	514 (36.4)	151 (36.5)	424 (23.1)	< 0.001
Osteoarthr -itis/ -osis	Present	1	95 (2.6)	54 (3.8)	15 (3.6)	26 (1.4)	< 0.001
Osteo -penia / -porosis	Present	1	78 (2.1)	39 (2.8)	8 (1.9)	31 (1.7)	0.104
Fibromyalgia / fibrosis	Present	1	2551 (69.6)	869 (61.6)	273 (65.9)	1409 (76.7)	< 0.001
Diagnoses (reports)							
MRI disc pathology	Absent	0	353 (9.6)	121 (8.6)	37 (8.9)	195 (10.6)	0.089
	Mild	1	1974 (53.9)	763 (54.1)	209 (50.5)	1002 (54.5)	---
	Moderate	2	921 (25.1)	365 (25.9)	107 (25.8)	449 (24.4)	---
	Severe	3	415 (11.3)	162 (11.5)	61 (14.7)	192 (10.4)	---
MRI spinal canal stenosis	Absent	0	1495 (40.8)	545 (38.6)	156 (37.7)	794 (43.2)	< 0.001
	Mild	1	861 (23.5)	331 (23.5)	79 (19.1)	451 (24.5)	---
	Moderate	2	628 (17.1)	249 (17.6)	90 (21.7)	289 (15.7)	---
	Severe	3	679 (18.5)	286 (20.3)	89 (21.5)	304 (16.5)	---
MRI lateral recess stenosis	Absent	0	1954 (53.3)	711 (50.4)	206 (49.8)	1037 (56.4)	< 0.001
	Mild	1	626 (17.1)	270 (19.1)	57 (13.8)	299 (16.3)	---
	Moderate	2	702 (19.2)	267 (18.9)	92 (22.2)	343 (18.7)	---
	Severe	3	381 (10.4)	163 (11.6)	59 (14.3)	159 (8.7)	---
MRI foraminal stenosis	Absent	0	922 (25.2)	328 (23.2)	100 (24.2)	494 (26.9)	0.015
	Mild	1	976 (26.6)	373 (26.4)	93 (22.5)	510 (27.7)	---
	Moderate	2	1009 (27.5)	398 (28.2)	123 (29.7)	488 (26.6)	---
	Severe	3	756 (20.6)	312 (22.1)	98 (23.7)	346 (18.8)	---
MRI facet joint	Absent	0	722 (19.7)	249 (17.6)	89 (21.5)	384 (20.9)	0.055
	Mild	1	1707 (46.6)	659 (46.7)	181 (43.7)	867 (47.2)	---
	Moderate	2	687 (18.8)	266 (18.9)	80 (19.3)	341 (18.6)	---
	Severe	3	547 (14.9)	237 (16.8)	64 (15.5)	246 (13.4)	---
MRI sacroiliac joint	Present	1	618 (16.9)	258 (18.3)	75 (18.1)	285 (15.5)	0.086
MRI endplate	Present	1	1615 (44.1)	647 (45.9)	183 (44.2)	785 (42.7)	0.201
MRI olisthesis	Present	1	1151 (31.4)	460 (32.6)	137 (33.1)	554 (30.1)	0.241
MRI curvature	Present	1	870 (23.8)	364 (25.8)	105 (25.4)	401 (21.8)	0.022
MRI fracture	Present	1	99 (2.7)	35 (2.5)	12 (2.9)	52 (2.8)	0.804

For patients with radiology reports (*n* = 3,663), patient profiles were derived from both clinical chart (demographics, psychosocial, and diagnoses) and radiology report (mri_diagnosis). Categorical patient profile values were mapped to an integer “ID” for downstream modeling and interpretation. Descriptive statistics report the count and percentage prevalence of each value overall and stratified by medication outcome. Statistical comparisons across medication classes were conducted using chi-squared tests with Bonferroni correction for categorical and binary features, and one-way ANOVA for numerical features. P-values < 0.05 indicated statistical significance in NSAID and opioid prescriptions among demographics (*age at first imaging*,* year at first imaging*,* obesity*), psychosocial determinants (*race/ethnicity*,* smoking status*,* social support*,* primary insurance*,* history of anxiety or depression*), medical chart diagnoses (*LBP duration*,* LBP laterality*,* numbness / tingling*,* radiculopathy disc pathology*,* spinal stenosis*,* facet joint arthropathy*,* sacroiliac joint*,* sciatica*,* osteoarthritis / osteoarthrosis*,* osteopenia / osteoporosis*,* fibromyalgia*), and radiology report diagnoses (*disc pathology*,* spinal canal stenosis*,* lateral recess stenosis*,* foraminal stenosis*,* curvature*). Significant associations in medication from clinical chart derived features are consistent across patient profiles comprised of only clinical chart features (*n* = 4,077).

### Evaluation of tree-based classifiers modeling medication prescription practices

**Fig. 2:**
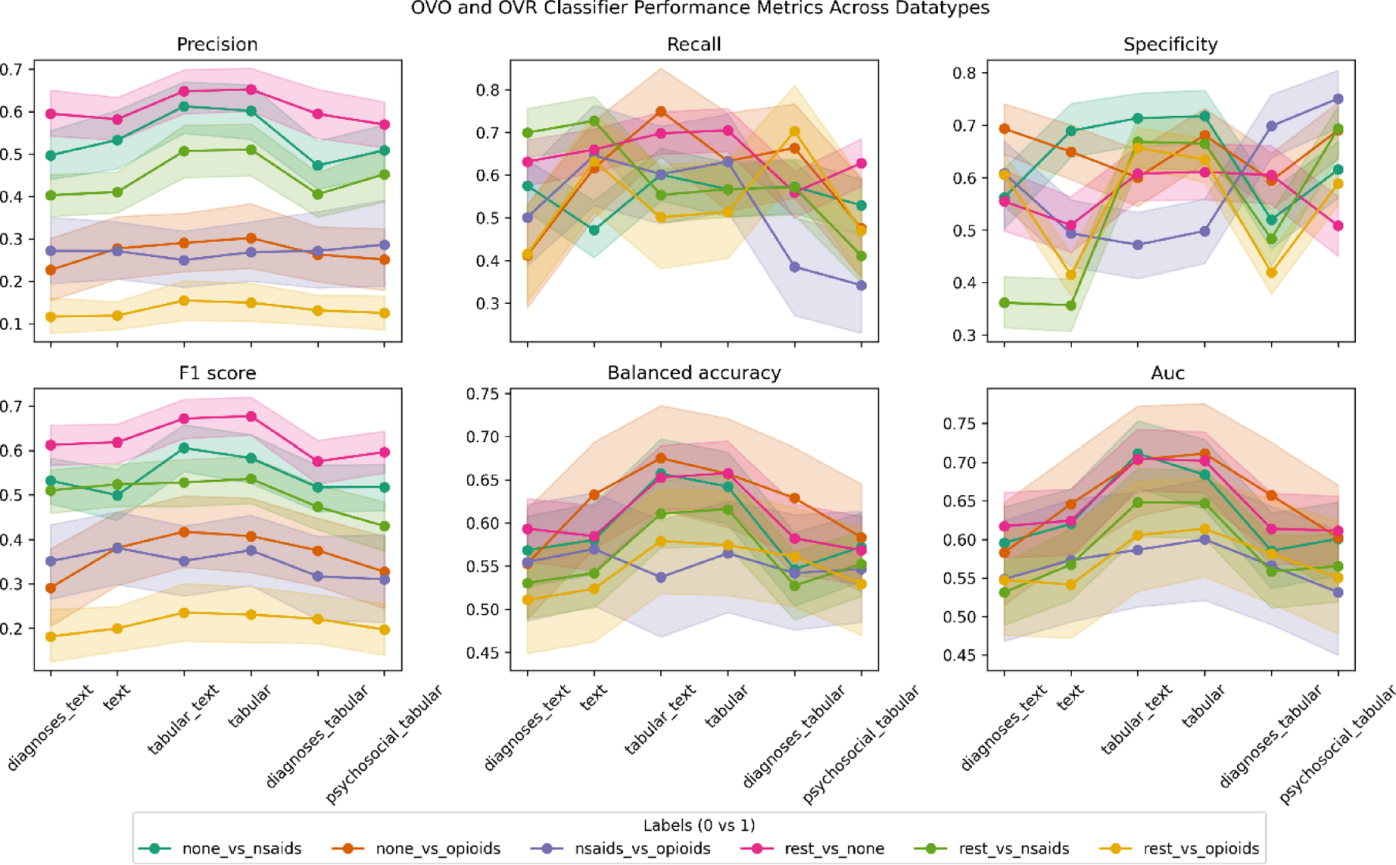
Parallel coordinates plot.

In total, 36 finetuned models predicted medication for patients with ns-cLBP across six feature subsets (*datatype*) and six medication class comparisons (*outcome*) with optimal hyperparameters listed in *Supplemental Table 3*. Bootstrapped test-set performance metrics and 95% confidence intervals are presented in a parallel coordinates plot (Fig. 2) and *Supplemental Table 4*.

Parallel coordinates plot of test-set performance metrics for 36 finetuned models, with 95% confidence intervals from bootstrapping (*n* = 1000). Each color represents a one-vs-one or one-vs-rest classifier to compare relative differences in medication classes (*none_vs_nsaids, none_vs_opioids, nsaids_vs_opioids*) and the likelihood of a specific class (*rest_vs_none, rest_vs_nsaids, rest_vs_opioids*) for non-specific chronic LBP treatment. The x-axis specifies patient profile compositions (datatype), detailed in Fig. 1 (e), with the most comprehensive profile (*tabular_text*) positioned near the center. Overall, models distinguishing opioid prescriptions (*none_vs_opioid, nsaid_vs_opioid, rest_vs_opioid*) exhibited lower precision suggesting greater variability in opioid usage, although this trend did not persist over recall and balanced accuracy metrics. Models incorporating clinical chart-derived diagnoses achieved the highest balanced accuracy, particularly for certain classifications (*none_vs_opioids, none_vs_nsaids, rest_vs_none*). Social determinants of health improved predictive performance when combined with pathological diagnoses but performed relatively poorly in isolation. These findings emphasize the dominant role of pathology in medication classification and the moderate contribution of social determinants.

### Performance metrics assessment

Bidirectional (OVO: *none_vs_nsaids*,* none_vs_opioids*,* nsaids_vs_opioids*) and class-specific (OVR: *rest_vs_none*,* rest_vs_nsaids*,* rest_vs_opioids)* comparisons shown no dominant strategy, mirroring real-world challenge of tailoring prescriptions to individual patient needs. Note *rest* labels encompass the mathematical complement of the one label, for example *rest_vs_none* compares NSAID or opioid to no medication. Predictive performance was quantified using the area under the curve (AUC) (mean [min, max] = 0.62, [0.53, 0.71]) and balanced accuracy (mean [min, max] = 0.58, [0.51, 0.68]), ensuring a thorough assessment of discrimination. Additionally, precision (mean [min, max] = 0.39, [0.12, 0.64]), recall (mean [min, max] = 0.53, [0.06, 0.75]), specificity (mean [min, max] = 0.64, [0.41, 0.98]), and F1-score (mean [min, max] = 0.42, [0.09, 0.65]) offered a comprehensive evaluation on classification performance. In relation to other clinical applications^29^, AUC and balanced accuracy values suggest weak (0.50–0.60) to acceptable (0.60–0.80) performance. Results reflect the inherent complexity of medication prescription patterns for non-specific chronic LBP patients and provide an initial benchmark for future investigations.

### Comparative analysis of multi-modal documentation sources for patient profiles

Performance trends across feature subsets revealed that documentation method for diagnoses influenced prescription prediction differently, when demographic and psychosocial features were held constant. Models trained with diagnoses from clinical charts (*tabular*) outperformed baseline models trained with all documented diagnoses (*tabular_text*) by 3% (SD = 7%) across all six pairwise outcomes and all six metrics, meaning the contribution of imaging-confirmed pathology marginally reduced predictive power. Furthermore, models trained with diagnoses from MRI-interpreted radiology reports (*text*) underperformed by 3% (SD = 16%) compared to baseline models (*tabular_text*), indicating imaging pathology alone provided less consistent information for prescription discrimination.

### Comparative analysis of biopsychosocial patient profiles

When isolating biopsychosocial effects on medication prescriptions with demographics held constant, ablating psychosocial determinants from clinical features (*tabular_diagnoses*) reduced performance only modestly by 4% (SD = 11%), while removing diagnoses (*tabular_psychosocial*) incurred a larger 11% (SD = 14%) reduction. This asymmetry implies diagnostic features primarily but not exclusively influence decision making. Nonetheless, diagnosis-free models leveraging demographics and psychosocial factors (*tabular_psychosocial*) achieved 53–59% balanced accuracy across OVO and OVR outcomes, underscoring how social determinants of health partially mitigate diagnostic uncertainty in real-world treatment pathways.

### Feature importance assessment and rank aggregation

**Fig. 3:**
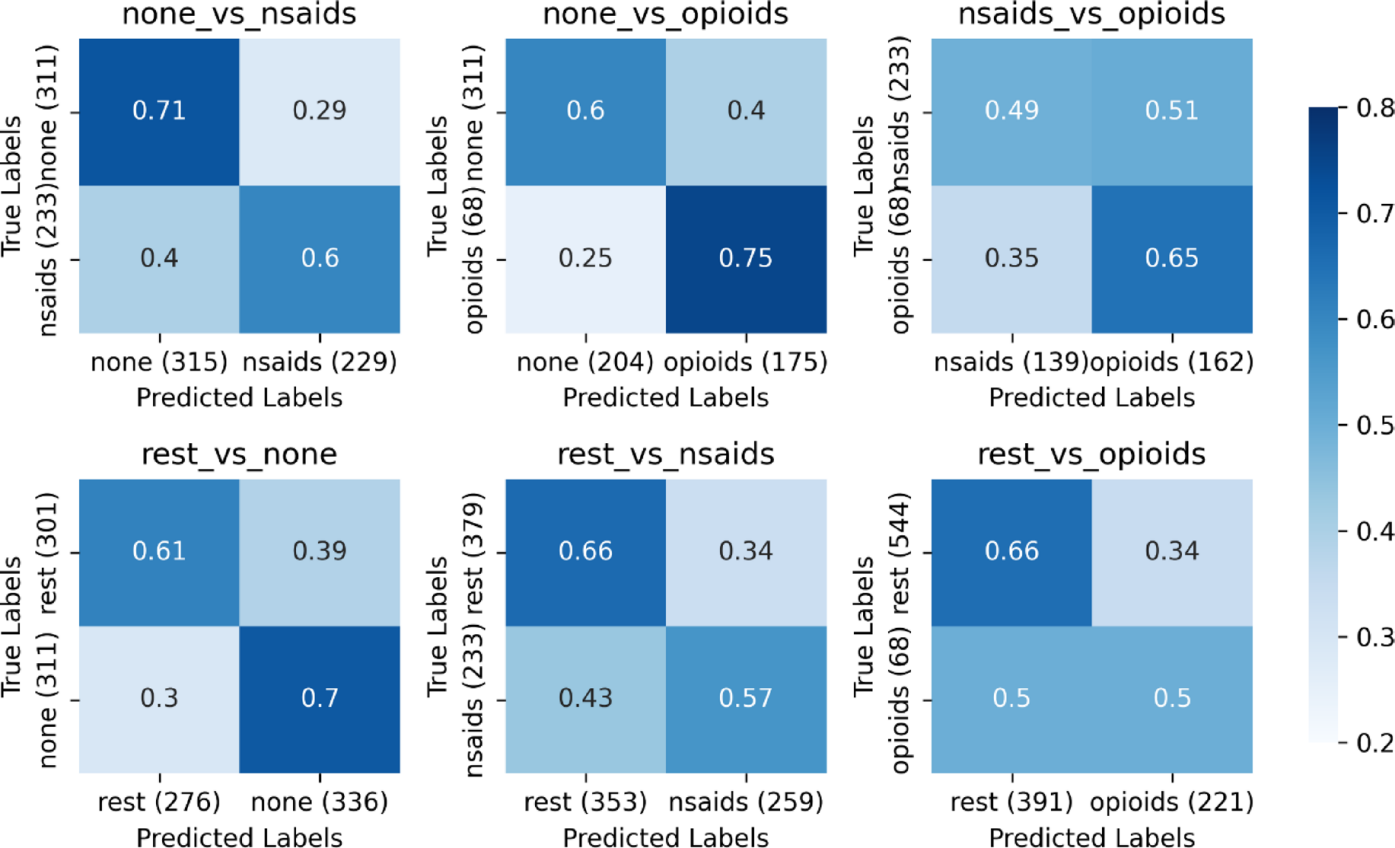
Normalized confusion matrices.

**Fig. 4:**
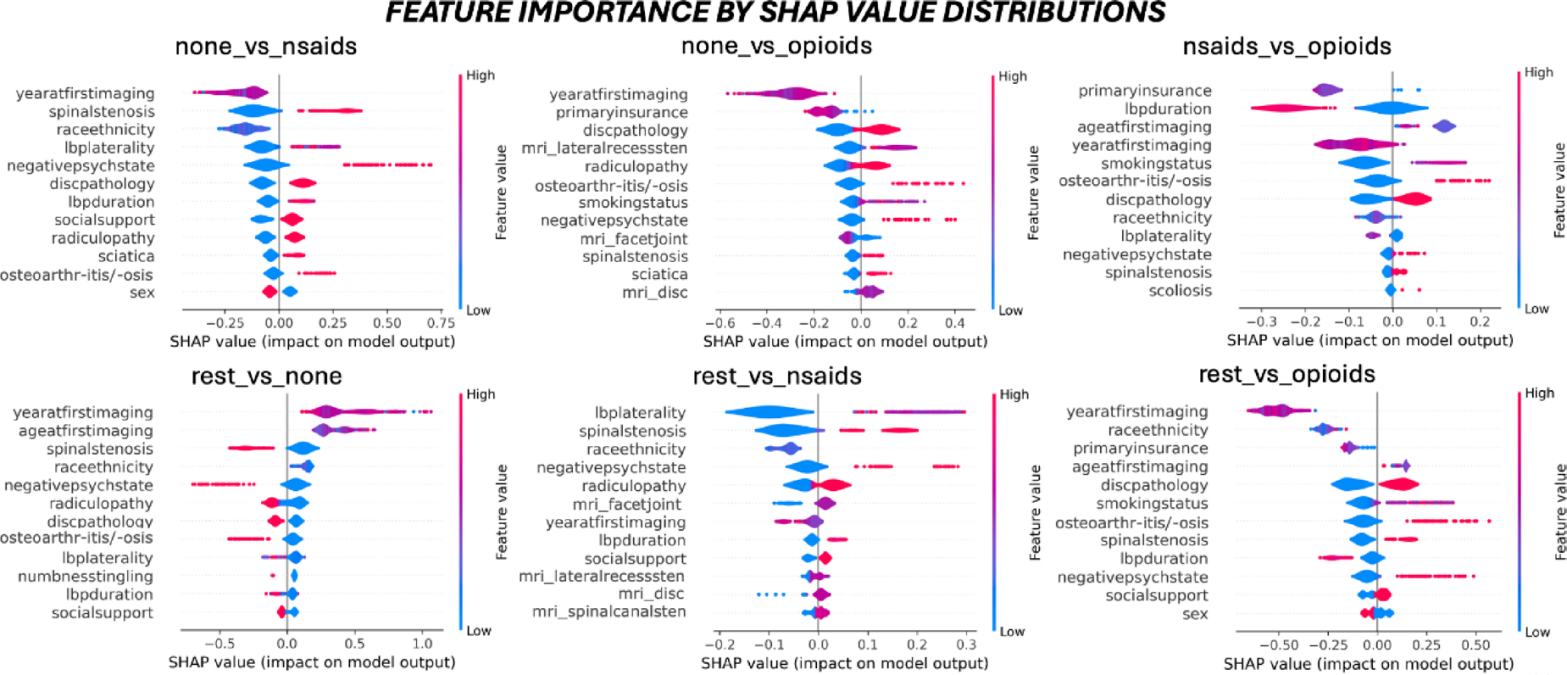
SHAP global feature importance.

Feature importance extraction utilized the best performing models in Fig. 3, which presents normalized confusion matrices for performance and misclassification context. True positive rates exceeded chance for all classifications except *rest_vs_opioids* (TPR = 0.53) and false positive rates were below chance for all classifications except *nsaids_vs_opioids* (FPR = 0.48), potentially reflecting clinical shifts towards reducing opioid prescription. SHAP violin plots in Fig. 4 rank feature importance in descending order and illustrate each feature’s influence (*SHAP_value*) on the likelihood of predicting the event class (*reference_vs_event*). Feature rankings are quantified in Supplemental Fig. 1.

Normalized confusion matrices for the best-performing models across medication classification tasks (*reference_vs_event*). Class labels were assigned so that the event label (1) corresponds to higher medication potency, except for *rest_vs_none* when the event represents no medication prescription. Matrices display prediction distributions, with diagonal elements representing correct classifications, off-diagonal elements showing errors, and sample sizes specified in the axis label. Models generally achieve moderate discrimination with true positive rates (TPR, lower right) exceeding false positive rates (FPR, upper right), indicating reliable classification with minimal false alarms despite a few exceptions. *Rest_vs_opioids* exhibit a low TPR and FPR, suggesting conservative prediction criteria that miss some opioid prescriptions. *Nsaids_vs_opioids* exhibits high TPR but FPR of 0.48, indicating the model captures opioid prescriptions but generates many false positives, potentially due to reductions in prescriptions to curb the opioid epidemic.

SHAP (SHapley Additive exPlanations) values quantify the relative importance of patient profile features for each one-vs-one (OVO) and one-vs-rest (OVR) medication prediction task. SHAP violin plots illustrate the distribution and directionality (positive / negative) of feature effects on model predictions, where the width reflects observation frequency, x = 0 indicates no impact on likelihood, and the colorbar corresponds to the values in Table 1. Overlapping SHAP distributions (e.g., *year_at_first_imaging*,* race_ethnicity*,* primary_insurance*) suggest complex decision boundaries, while distinct distributions whether symmetric (e.g., *disc_pathology*) or skewed (e.g., *negative_psych_state*) indicate more deterministic relationships. Presence of spinal pathologies (*disc*,* stenosis*,* sciatica*) and *social_support* were consistently associated with higher drug classes.Longer *LBP_duration* correlated positively with NSAIDs and negatively with opioids while documenting either unilateral or bilateral *LBP_laterality* correlated positively with NSAIDs compare to no specification. Female sex (0) was linked to a higher likelihood of medication (*none_vs_nsaids*,* rest_vs_opioids*) although the magnitude of effect was relatively small compared to other patient characteristics. These insights suggest complex interactions between clinical and sociodemographic factors in medication decision-making for non-specific chronic lower back pain.

### Key predictors of prescription patterns by SHAP value magnitude

Temporal and pathological features consistently emerged among the most influential predictors of medication prescription pathways. Most notably, *year_at_first_imaging* averaged the highest SHAP value magnitudes across most models, suggesting a strong association with shifts in opioid prescription practices following guideline changes. In contrast, *spinal_stenosis*, *disc_pathology*, *negative_psych_state*, *osteoarthritis_osis* and *race_ethnicity* showed variable importance depending on the classification task, underscoring potential interactions between patient characteristics and prescription decisions.

### Features impact on prescription likelihood by SHAP value directionality

For pathology-related features, the presence of spinal stenosis, disc pathology, radiculopathy, sciatica, lateral recess stenosis, spinal canal stenosis, sacroiliac joint pathology, endplate changes, and osteoarthritis / osteoarthrosis all increased the likelihood of stronger medication prescriptions. Interestingly, facet joint arthropathy and increased the likelihood of a NSAID prescription (*rest_vs_nsaid*) but decreased the likelihood of an opioid (*none_vs_opioid*). The same effect was observed for specification of LBP duration (chronic or acute, vs. unspecified) and LBP laterality (unilateral or bilateral, vs. unspecified) (*none_vs_nsaid*,* nsaid_vs_opioid*,* rest_vs_none*,* rest_vs_nsaid*,* rest_vs_opioid*).

Among psychosocial factors, *year_at_first_imaging* consistently reduced the likelihood of any or escalated medication. However, the effect of earlier or later years were unclear due to complex value distribution patterns. In contrast, female patients or those with anxiety or depression consistently associated with stronger medication than their counterparts. Furthermore, patients having a partner were more likely to receive some prescription than those who were single or separated (*none_vs_nsaid*,,* rest_vs_none*,* rest_vs_nsaid*,* rest_vs_opioid*). *Sex* and *social_support* demonstrated opposing and symmetrically distributed effects on classification tasks, while *negative_psych_state* and *smoking_status* had opposing and skewed effects with negative perceptions exerting stronger influence. These findings provide insight into clinical decision making and reinforce the importance of incorporating biopsychosocial patient information when modeling prescription patterns.

### Rank aggregated features for all medication prescription decisions

Feature importance across all bi-directional (OVO) and class-wise (OVR) medication prescription decision are summarized in Table 2. Rank aggregation analysis revealed that temporal factors, specifically *year_at_first_imaging*, demonstrated the strongest association with medication prescriptions across all model variants, consistent with substantial updates to prescribing guidelines over the past decade.

**Table 2:** Rank aggregated feature importance.

Top ten features for				
	Best models	*tabular_text* models	*tabular* models	*text* models
1 2 3 4 5 6 7 8 9 10	year_at_first_imaging race_ethnicity **spinal_stenosis** negative_psych_state **mri_disc** **radiculopathy** **mri_facet_joint** **lbp_laterality** primary_insurance **disc_pathology**	year_at_first_imaging **spinal_stenosis** race_ethnicity negative_psychstate **radiculopathy** **lbp_duration** **lbp_laterality** **osteoarthritis_osis** **mri_facet_joint** **mri_lr_stenosis**	year_at_first_imaging race_ethnicity **spinal_stenosis** age_at_first_imaging primary_insurance **lbp_duration** **disc_pathology** negative_psych_state **osteoarthritis_osis** **lbp_laterality**	year_at_first_imaging race_ethnicity **mri_disc** **mri_facet_joint** primary_insurance **mri_lr_stenosis** negative_psych_state **mri_sc_stenosis** smoking_status **mri_f_stenosis**

Feature importance rankings were aggregated across all one-vs-one (OVO) and one-vs-rest (OVR) medication prescription classification tasks using variational Borda Count methodology. The top ten predictive features are presented for models incorporating: (1) the highest balanced accuracies, (2) the complete predictor dataset, (3) *tabular* predictors excluding text-derived features (*mri_“diagnosis”*), and (4) *text* predictors excluding tabular-derived diagnoses (*“diagnosis”*). Physiological features are bolded with psychosocial determinants are not. Rankings reveal key associations between patient characteristics and prescriptions for non-specific chronic lower back pain, within the constraints of finetuned model performance. Abbreviations are as follows: lbp – lower back pain, lr_stenosis – lateral recess stenosis, sc_stenosis – spinal canal stenosis, f_stenosis – foraminal stenosis.

Highly ranked features were fairly evenly distributed between physiological and social determinants of health, reflecting alignment with multifaceted prescribing recommendations. *Race_ethnicity* (high importance), *negative_psych_state* (low importance), and *primary_insurance* (low importance) demonstrated consistent associations, suggesting potential disparities in opioid prescription, although specific class associations were not analyzed. Among pathology features, *LBP_laterality*, *disc_pathology*, and *spinal_stenosis* ranked prominently, corroborating other findings in this study.

Through comparative analysis of diagnoses documentation sources (*tabular_text*), clinician-documented pathologies in structured clinical charts (*stenosis*,* radiculopathy*) showed stronger associations than their counterparts extracted from radiology report (*mri_spinal_canal_stenosis*,* mri_lateral_recess_stenosis*). However, certain radiological findings, specifically facet joint and lateral recess pathology (*mri_facet_joint*,* mri_lateral_recess_stenosis*), were uniquely predictive when extracted from imaging reports. Additionally, chart-documented pain descriptors (*LBP_duration*,* LBP_laterality*) demonstrated high predictive value despite offering less detailed and standardized information than formal questionnaires.

## Discussion

This study is the first to systematically evaluate ML models for predicting medication prescriptions in patients with ns-cLBP, leveraging multimodal EMR data that spans biopsychosocial features. By applying OVO and OVR models with SHAP interpretation, we offer a transparent approach to understand the complex interplay of pathological, psychosocial, and demographic factors influencing prescription decisions. Our findings establish a benchmark on who is currently prescribed medications for ns-cLBP and demonstrate the potential of explainable AI to characterize decision-making in complex conditions like pain management. This framework can serve as a practical institutional monitoring tool to identify characteristics associated with pharmacologic interventions, misalignment between treatment pathway predictors and established guidelines, and underserved target populations for future resource allocation and policy changes.

Tree-based models were selected because they capture non-linear interactions among multiple predictors more effectively than traditional logistic regression, while maintaining interpretability and achieving performance comparable to deep learning models^30–32^. Despite model complexity and predictors curated from clinically-relevant EMR^2,8,10^, our models showed only weak to acceptable discrimination (AUC: 0.53–0.71; balanced accuracy: 0.51–0.68). Our model underperformed compared to general opioid prescription and misuse models (AUC: 0.93–0.94)^28^. This performance gap likely reflects fundamental differences in study design and priorities. While we focused on ns-cLBP patients across all care settings and prioritized model interpretability, the high-performing opioid models targeted a more homogeneous hospitalized population and incorporated broader patient features including laboratory results and clinical note embeddings using transformer model architectures. Although these design choices enhance predictive accuracy, they sacrifice the clinical interpretability essential for understanding prescribing patterns.

Our model performance was comparable to existing models that predict 10–12 week prognosis for patients with chronic musculoskeletal pain or new onset LBP, using patient questionnaire responses on pain (location, intensity, duration, interference), mobility (walking distance, hours of paid work), and psychological factors (cognitive flexibility, depression) (AUC: 0.49–0.71)^33,34^. These results reflect nuanced decisions about biopsychosocial factors involved in medication prescription for LBP^19^ and chronic pain management which is challenging for practitioners in 14% of encounters^35^. Modest performance suggest that prescription decisions are driven by diverse factors not fully captured by this study, including detailed pain descriptors and possible NSAID co-medication known to prescribing physicians but not documented in the EMR^36^. Nevertheless, even modest prediction accuracy can inform retrospective analyses of prescribing trends and guide prospective interventions to align practices with evolving guidelines.

Quantifying model performance through machine learning revealed unexpected limitations in the contribution of radiological information towards medications for ns-cLBP. Integration of “*tabular_text*” features reduced prediction performance by 3% with high variability (SD = 7%) compared to models using “*tabular*” data alone, while exposing substantial disparities in diagnostic documentation (κ = 0.102 ± 0.0183 with 2–9 times higher frequencies in tabular medical charts). The low prevalence of diagnoses in radiology reports may be attributed to radiologists’ limited knowledge of symptoms during reporting^37^,time limitations, and liability considerations. Meanwhile diagnoses from medical charts span the patient’s entire history and physicians can ask about patient symptoms. These findings align with evidence-based guidelines that advocate for the selective use of conventional imaging, given that MRI findings often correlate poorly with current or future LBP^12^ and can contribute to increased healthcare utilization without clear benefits^14,15^. Prescribing patterns alongside prior research on perceived benefits of diagnostic imaging^38^ suggest that currently medications may target pain perception and suspected pathology more so than image-confirmed pathology.

When assessing biopsychosocial factors contribution to “*tabular*” model performance using ablation techniques, “*diagnoses*” contributed 11% to performance metrics followed by “*psychosocial*” features at 4%, with notable dependence on specific metrics indicated by large standard deviations (11–14%). The limited relative contribution of psychosocial features may be partly due to sparse documentation in de-identified EMRs, which lack the depth of patient-reported questionaries or psychosocial narratives in clinical reports present in other studies^39,40^. These findings reinforce the need for comprehensive patient assessments in chronic pain management, where prescription decisions extend beyond pathology to include psychological and social influences. However, challenges remain for clinicians in determining how to allocate time when treating patients with chronic pain^41^, as these cases often require prolonged management and image-derived pathology may not always provide clear clinical guidance.

The strong influence of temporal features, particularly “*year_at_first_imaging*”, aligns with measurable reductions in opioid prescription practices following updated clinical guideline aimed at curbing the opioid epidemic^5,6,42^. Our results suggest that pathology-driven prescriptions remain dominant, as structural abnormalities were consistently associated with stronger medications. This aligns with a meta-analysis that found spinal stenosis and disc degeneration had the highest probability of being related to LBP^7,12^. The higher feature importance of stenosis compared to radiculopathy may reflect differences in baseline prevalence (15–38% of patients seeking care^43^ vs. 5–10% of LBP patients^44^ ), documentation patterns (consistency reporting severe stenosis vs spectrum of radiculopathy severity), imaging visibility (canal narrowing is more identifiable than nerve root compression), and available non-surgical treatment alternatives (stenosis patients may escalate to stronger medications earlier while radiculopathy patients often respond well to paraspinal injections, nerve blocks, and physical therapy^44)^. Notably, “*facet_joint_pathology*” was associated with NSAID use but not opioids, potentially reflecting differences in clinical management strategies for mechanical versus inflammatory pain sources. These findings highlight the ongoing impact of evolving clinical guidelines on prescribing trends and the role of pathology in shaping treatment decisions.

The observation that specific LBP laterality and duration increased the likelihood of stronger prescriptions may reflect clearer symptom communication and enhanced patient-physician alignment. Well-defined symptom presentations indicate effective communication, including clearer treatment plans and expectations, which are strongly associated with improved patient satisfaction and health outcomes compared to low-agreement settings^45,46^. When patients present with specific, well-characterized symptoms, clinicians and patients may more appropriately select interventions and establish realistic expectations. These observations emphasize the importance of comprehensive symptom assessment in clinical decision-making and warrant future investigations incorporating questionnaire-based pain descriptors.

Prior research has also highlighted the role of race, ethnicity, and psychological state in opioid prescribing patterns and LBP, despite challenges in defining social determinants of health^47,48^. In this study, the variable importance of demographic and psychosocial factors highlights context-dependent prescribing behaviors, supporting a more nuanced, patient-specific approach. The association between female “*sex”* and stronger medication prescriptions raises questions about potential gender biases in pain management, warranting further investigation. This aligns with prior studies that found higher chronic LBP prevalence among females^47^ and higher healthcare costs for smokers within a year after LBP presentation^49,50^, but challenges the finding that men incur high healthcare costs^49,50^.

Similarly, we found the SHAP directional influence of “*smoking_status*” indicated that current and former smokers were more likely to receive opioids but not NSAIDs although the predictive power was weak. The association in a “*negative_psych_state*” and stronger prescriptions, along with the link between “*social_support*” (being partnered rather than single/separated) and increased medication use, suggests that clinicians consider psychosocial distress when prescribing. This prescription pattern is consistent with previously found associations in depression and being married / windowed / separated / divorced increased chronic LBP prevalence^47^. These findings underscore the need for integrated pain management strategies that extend beyond pharmacologic interventions to incorporate evidence-based approaches such as cognitive-behavioral therapy, physical therapy, and self-management education consistent with prior work^5,9–11^.

Altogether, these findings offer healthcare institutions with an actionable framework to systematically evaluate their ns-cLBP management practices and identify opportunities for guideline-concordant care. The therapeutic void in managing non-specific, chronic pain is reflected in low model precision (mean: 0.39) despite using expert-curated LBP patient profiles, which underscores the lack of consensus on pharmacological interventions. Nonetheless, data-driven insights provide a starting point for further intervention. Three specific areas of focus target conservative opioid prescriptions, reducing imaging overutilization, and encouraging biopsychosocial patient assessments. While educational efforts had no effect on the rate of low-value spinal imaging rates^51^, communicating benchmark prevalence metrics of normal MRI findings improved guideline concordance through decreased subsequent imaging^52^, opioid prescription^53^, and patient perceived severity of spinal pathology^54^. Similarly, clinicians and researchers can use global SHAP-based feature importance metrics as an accessible, data-driven reference to compare real-world prescribing patterns against guideline-specific risk indicators. Comparisons provide feedback on incorporation of the biopsychosocial model into treatment planning and identify target populations that may benefit from focused interventions.

The interpretation of our findings was constrained by several limitations of the EMR database. The database lacked patients’ primary complaints, prescribing rationales, and treatment response metrics that typically guide clinical decision-making. These limitations were mitigated through clinically informed selection criteria and the characterization of prescription patterns instead of prescription efficacy, which varies with individuals’ pain perception including neuropathic, nociceptive, and nociplastic subtypes^55^. Our findings require comparison with other studies to assess prescription appropriateness and cannot be used prospectively. Future research may address these gaps by refining the outcome variable to include prescription details (e.g. frequency, duration, dosage), prescription adjustments over time, prescription department, and patient-reported outcomes (e.g. pain scores, days lost from work, disability adjusted life years). This comprehensive approach could offer a holistic understanding of treatment decision-making and help identify low-efficacy prescriptions, which remains poorly understood for ns-cLBP patients.

LBP patient profiles were also limited to characteristics available in our EMR database. The cohort contained only patients who had a lumbar spine MRI between 2012 and 2024 in San Francisco, California. Thereby we excluded broader LBP populations that differ by imaging, timeframe, or location despite over-utilization of imaging compared to clinical guidelines (31–54%)^5^. Additionally, we did not require comprehensive chart reporting or image annotations to reflect standard clinical practice, which may have obscured detection of infrequently documented diagnoses with emerging value (i.e. endplate changes) or imaging insights. Other variables unavailable in our de-identified EMRs for future investigation include patient weight, BMI, pain descriptors, and socioeconomic status. Standardizing comprehensive EMR documentation of pain characteristics and treatment outcomes would better capture biopsychosocial factors relevant to pain management and facilitate development of advanced prognostic assessment tools beyond current monitoring capabilities.

While tree-based models were prioritized for interpretability, emerging ML architectures could better model multimodal biopsychosocial interactions. Future modeling investigations should pursue developing standardized reporting of quantitative imaging measures^56^, investigating advanced natural language processing techniques that can better interpret unstructured reports, and exploring direct image encoding to bypass documentation limitations altogether. Future studies may integrate longitudinal patient data, raw data such as through latent space encoding or quantitative imaging^57,58^, and validate models prospectively in diverse care settings. Feature importance is constrained by historical patterns that may encode regional and institutional biases, such that emerging clinically relevant features on frequently documented ones.

In conclusion, this study provides an analytical framework to characterize medication prescription decisions in ns-cLBP using multimodal patient profiles. While performance metrics highlight the inherent uncertainty in treating this challenging condition, our findings illuminate addressable patterns in current prescribing practices and demonstrate the promise of multi-modal data integration in chronic pain management algorithms. This approach offers a valuable tool for monitoring clinical decision-making, as guidelines related to imaging, psychosocial factors, and opioid use evolve. This transferable pipeline can be applied to diverse datasets with SHAP feature importance adapted to local patient populations, enabling comparisons across institutions and time periods. By identifying prescription pattern misalignments that may reveal systematic biases in pain management, these analyses enable healthcare systems to implement corrective measures toward more consistent, equitable, and personalized care strategies.

## Methods

All research procedures were conducted in accordance with relevant guidelines and regulations. Under the UCSF Institutional Review Board’s guidance, IRB review was not required due to the use of de-identified data.

### Cohort curation

#### Cohort selection

UCSF’s de-identified “Info Commons” database enabled the integration of large multi-modal clinical datasets for retrospective analysis in accordance with relevant guidelines^59^. Strict selection criteria in Fig. 1 (a) minimized LBP subtype heterogeneity while ensuring a representative cohort to reduce the likelihood to identifying characteristics for well characterized LBP subtypes.

- **Inclusion criteria: **Eligible patients underwent lumbar spine MRI without contrast between 2012 and 2024. MRI facilitates practical visualization for spinal pathology assessment and is typically performed for (A) chronic, unresolved pain or (B) suspected treatable pathology
- **Exclusion criteria: **To maintain focus on unresolved pain cases (chronic or significant enough to warrant imaging), the study excluded patients with prior spinal surgery or paraspinal injections, which often indicate specific, operable conditions. Additional exclusions removed patients with fracture, cancer, infection, trauma, or stroke were to eliminate red flags symptoms that suggest a more serious underlying pathology. Patients younger than 18 were excluded due to fundamental differences psychosocial states and prescription practices between adult and pediatric populations.

#### Multi-modal patient health profile definition

- **Outcomes: **Viable metrics were carefully considered since clinical data lacks healthy controls to allow for meaningful interpretation. To investigate non-invasive strategies for ns-cLBP management, outcome variables were defined as the most potent medication received within one year of the lumbar spine MRI where levels are defined as none, NSAIDs, or opioids^5,11,13,42,60,61^ in Fig. 1 (b). Specific selection criteria are included in Supplemental Code Snippet 1.
- **Predictors: **Patient profiles were constructed by concatenating biopsychosocial factors from multi-modal documentation sources up to one month after their first lumbar spine MRI. Demographics, psychosocial determinants, and diagnoses were extracted from medical charts, while imaging-confirmed diagnoses were extracted from radiology reports. Patient profile characteristics are summarized in Fig. 1 (c) and Supplemental Table 5, while the extraction pipeline is detailed in Fig. 1 (d).

### Cohort extraction

We considered the complexity, quality, and consistency of each data type to motivate an extraction strategy for clinically informed patient profiles. Tabular medical charts offer structured diagnostic and psychosocial data^47,48^, MRIs provide visualization of tissues and quantitative metrics, and radiology reports offer nuanced image insights from expert annotations. To streamline feature extraction in alignment with expert clinical assessments, we derive imaging features associated with LBP directly from annotated radiology reports, bypassing the need for intermediary model development on MR images.

#### SQL query design for LBP features from clinical charts

To create LBP patient profiles from tabular data, clinical chart information was mapped to each patient category using a one-to-one or many-to-one approach. One-to-one mappings were applied when there were a limited number of labels and patients only belonged to one label within a category with usefulness for demographic data (i.e. sex, race/ethnicity,...). Many-to-one mappings were defined for patient pathology to combine numerous labels together into meaningful categories. This data cleaning strategy required careful query design in collaboration with clinical experts to reconcile differences in medical terminology and maintain confidence in the label extraction (i.e. disc pathology encompasses herniated disc, disc degeneration, discogenic pain, ruptured lumbar disc, but not *disc*harge).

Missing demographic variables described in Supplemental Table 6 were assessed to determine the type of missingness and guide the selection of an imputation method. Little’s MCAR test was used to evaluate whether data were “missing completely at random” (MCAR), and logistic regression assessed whether data were “missing at random” (MAR) based on a binary missingness indicator (*y*_*miss*) (Supplemental Table 7). After initial analyses determined the data were “missing not completely at random” (MNAR), attempts to identify auxiliary variables that could account for the missingness pattern and render the data MAR were unsuccessful. As a result, maximum likelihood imputation using the expectation-maximization based KNNImputer was applied to infer missing values for *race_ethnicity* (6.5%), *social_support* (8.3%), *smoking_status* (12.7%), and *primary_insurance* (32%), using the features: *sex*,* age_at_first_imaging*,* year_at_first_imaging*, and *preferred_language*.

#### Foundational models for human-interpretable pathology detection from radiology reports

We adopted a target approach to create patient-feature vectors from multi-modal data by pre-selecting relevant MRI features to extract from radiology reports. An LLM data extraction pipeline VERSA-spine^62^ was used to summarize the presence of clinically relevant spinal pathology^7,8^ identified in radiology reports. This collection of prompts was run using GPT-4.0 on an internal PHI-compliant environment using an API key for $450. Since the algorithm was refined and validated on clinical reports from our institution, we did not anticipate difference in data distributions. Nonetheless, validation was performed on a subset of 20 notes with human-annotated labels for quality assessment (Supplemental Table 1) and consistency with clinical chart diagnoses was evaluated using Cohen’s kappa across all notes (Supplemental Table 2). Two minor prompt modifications were made to extract pathology with the granularity of mild / moderate / severe as opposed to absent / present, and provide prompt context that guides interpretation when pathology is not indicated.

#### Statistical testing

Prior to ML modeling, hypothesis testing identified significant differences in categorical medication outcomes across each patient profile predictor. Chi-squared test of independence was used for categorical predictors and one way ANOVA was used for continuous predictors. The threshold for statistical significance was set to *p* < 0.05.

### Predictive modeling

#### Classifier training

We implemented random forest^63^, bagging, adaboost^64^, and XGBoost^65^ tree-based classification algorithms to mathematically model medication prescription pattens by using rule-based splitting. Training incorporated 5-fold cross validation for robustness and grid-search hyperparameter finetuning for trees count, maximum depth, minimum samples per split, minimum samples per leaf, bootstrapping, and estimator count. The best models were selected based on highest validation balanced accuracy. Thirty-six models were finetuned across medication outcomes (*n* = 6) and patient profile predictor datatypes (*n* = 6) (Fig. 1 (e)).

Models predicted bidirectional one-vs-one outcomes (OVO: *none_vs_nsaids*,* none_vs_opioids*,* nsaids_vs_opioids*) and class-wise one-vs-rest outcomes (OVR: *rest_vs_none*,* rest_vs_nsaids*,* and rest_vs_opioids*) for no, NSAID, or opioid prescription. Following machine learning convention, we encoded event labels as “1” and reference labels as “0.” Clinically, the event represented stronger medication classes while the reference indicated lower medication classes, except when any medication served as reference relative to no prescription (*rest_vs_none*).

Patient profile vectors concatenated a combination of demographics, psychosocial determinants, and diagnoses from tabular clinical chart, and diagnoses from MRI radiology reports. The *tabular_text* set included all predictors, while variations limited diagnoses to be exclusively from clinical charts (*tabular*) or radiology reports (*text*). Health determinant stratification created subsets of demographics with psychosocial features from clinical charts (*tabular_psychosocial*), diagnoses from clinical charts (*tabular_diagnoses*), or diagnoses from radiology reports (*text_diagnoses*).

To evaluate ns-cLBP prescriptions, we assessed whether tree-based models could predict medication prescription. We then evaluated multi-modal documentation sources and biopsychosocial features by training classifiers with different feature combinations and comparing performance.

#### Model assessment and statistical analysis

Model evaluation on test-set data was assessed by precision, recall, specificity, F1-score, balanced accuracy, and AUC with bootstrapping (*n_bootstraps* = 1000, *n_samples* = length of *y_test*) to determine 95% confidence intervals from the 2.5-97.5th percentile range^66,67^.

We compared several types of patient profiles to assess their impact on model performance. Profiles containing diagnoses from medical charts (*tabular*) or radiology reports (*text*) were compared to a comprehensive profile that combined both sources (*tabular_text*). Additionally, profiles containing all available features from medical charts (*tabular*) were compared to profiles limited to pathological findings (*tabular_diagnoses*) and psychosocial determinants (*tabular_psychosocial*). We calculated the percent difference in performance metrics for each outcome and patient profile comparison using the formula:

$$\:percent\:diff=\frac{experimental-baseline}{baseline}\times\:100$$

Variability across patient profiles was then quantified by averaging the percent differences across all performance metrics and outcomes, and calculating the standard deviation.

### Feature importance

#### Explainable AI with SHAP values

Feature importance was determined using Shapley Additive exPlanations (SHAP) feature rank aggregation from the six best-performing models for each pairwise medication outcome Fig. 1 (f). Based on cooperative game theory, SHAP calculates the average marginal contribution of each feature on the model output, by measuring the positive or negative effect with and without the feature^25^. For EMR-based prediction models, the magnitude and directionality of SHAP values reveal which patient characteristics most strongly influence prescription recommendations, enhancing model interpretability and clinical trustworthiness.

#### Rank aggregated feature importantance for LBP prescriptions

To consolidate important features associated with LBP medication prescriptions, feature contributions were aggregated across OVO and OVR models using a variational Borda count method^68,69^. We quantified feature importance within each model by the mean SHAP value, ranked features with a mean SHAP value above 0.01 in descending order, assigned missing features a rank of zero, and normalized rankings to sum to one. This strategy leverages prior knowledge to assign and tolerate missing rankings (rank 0) unlike the traditional Borda count method. The final ranking summed normalized ranks across models, with higher totals indicating greater importance.

We performed rank aggregation for all best-performing OVO and OVR models to capture relative feature effects across interacting documentation sources (*tabular_text*), clinical chart features (*tabular*), and radiology report features (*text*). While SHAP values are not directly comparable across models, this rank aggregation approach effectively summarizes clinically relevant feature associations rather than absolute effect sizes which may be better represented through weighting terms. A ranked list provides a consolidated summary of feature importance. Downstream assessment will compare these findings with existing clinical care guidelines to better understand the discrepancy between guidelines and practice.

All analyses were performed using Python 3.11.9 on a Linux workstation running Red Hat Enterprise Linux release 9.5 (Plow) with an AMD EPYC 9354P 32-Core Processor CPU (64 threads) and 512 GB RAM. Analyses were conducted using duckdb (1.0.0), scikit-learn (v1.5.2), and shap (v0.46.0).

## Supplementary Information

Below is the link to the electronic supplementary material.

Supplementary Material 1

## Data Availability

Dataset access is available from the co-authors upon reasonable request.
