# Supplementary material for: Explainable AI reveals tissue pathology and psychosocial drivers of opioid prescription for non-specific chronic low back pain
Source: Sci Rep. 2025 Aug 21;15:30690. doi: 10.1038/s41598-025-13619-7 (PMC12370884; doi:10.1038/s41598-025-13619-7)
Supplement: Supplementary file 1 — Supplementary Material 1 [file 41598_2025_13619_MOESM1_ESM.docx]

Explainable AI reveals tissue pathology and psychosocial drivers of opioid prescription for non-specific chronic low back pain

Michelle W. Tong [1,2,3], Katharina Ziegeler [1], Virginie Kreutzinger [1], Sharmila Majumdar [1,3]

[1] Department of Radiology and Biomedical Imaging, University of California San Francisco, San Francisco, USA

[2] Department of Bioengineering, University of California Berkeley, Berkeley, CA 94720, USA

[3] Department of Bioengineering and Therapeutic Sciences, University of California San Francisco, San Francisco, USA

Table of Contents

[Supplementary Materials 2](#_Toc204862607)

[Supplemental Table 1: GPT Prompt Validation 2](#_Toc204862608)

[Supplemental Table 2: Tabular and Text Diagnoses Agreement 3](#_Toc204862609)

[Supplemental Table 3: Finetuned Model Hyperparameters 4](#_Toc204862610)

[Supplemental Table 4: Finetuned Model Test-Set Performance Metrics 7](#_Toc204862611)

[Supplemental Figure 1: SHAP Feature Importance Bar Plot 9](#_Toc204862612)

[Supplementary Table 5: Patient Profiles (clinical charts) 10](#_Toc204862613)

[Supplementary Table 6: Demographic Missingness 12](#_Toc204862614)

[Supplementary Table 7: Assessment of the Type of Missingness 13](#_Toc204862615)

[Code Snippet 1: 17](#_Toc204862616)

# Supplementary Materials

## Supplemental Table 1: GPT Prompt Validation

Validation of the LLM pipeline "Versa-Spine" occurred on 20 randomly selected radiology reports prior to extracting spinal pathology for the entire cohort. Performance metrics demonstrated F1-scores, false positive rates (FPR), false negative rates (FNR), and associated 95% confidence intervals (CI). These metrics supported acceptable data extraction performance, indicating a robust extraction prompt and limited data drift from the prompt development cohort, which included a different set of patients from the same institution.

| Pathology | F1 | F1: 95% CI | | FPR | FPR: 95% CI | | FNR | FNR: 95% CI | |
| --- | --- | --- | --- | --- | --- | --- | --- | --- | --- |
|  | **---** | **Lower** | **Upper** | **---** | **Lower** | **Upper** | **---** | **Lower** | **Upper** |
| Endplate Changes | **0.957** | 0.948 | 0.967 | **0** | 0 | 0 | **0.083** | 0.081 | 0.088 |
| Disc Pathology | **0.967** | 0.95 | 0.97 | **0.075** | 0.074 | 0.077 | **0.017** | 0.017 | 0.017 |
| Spinal Canal Stenosis | **0.933** | 0.925 | 0.943 | **0.042** | 0.042 | 0.043 | **0.034** | 0.034 | 0.036 |
| Facet Joint Arthropathy | **1** | 0.991 | 1.011 | **0** | 0 | 0 | **0** | 0 | 0 |
| Lateral Recess Stenosis | **0.905** | 0.901 | 0.91 | **0.022** | 0.022 | 0.022 | **0** | 0 | 0 |
| Foraminal Stenosis | **0.957** | 0.952 | 0.962 | **0.03** | 0.03 | 0.031 | **0.029** | 0.029 | 0.03 |
| Sacroiliac Joint | **1** | 0.953 | 1.053 | **0** | 0 | 0 | **0** | 0 | 0 |
| Olisthesis | **1** | 0.953 | 1.053 | **0** | 0 | 0 | **0** | 0 | 0 |
| Curvature | **0.889** | 0.847 | 0.936 | **0.091** | 0.084 | 0.101 | **0.111** | 0.101 | 0.126 |
| Fracture | **0** | 0 | 0 | **0** | 0 | 0 | **0** | 0 | 0 |
| Mean | **0.956 ± 0.041** | | | **0.029 ± 0.035** | | | **0.030 ± 0.041** | | |

Supplemental Table 2: Tabular and Text Diagnoses Agreement

Spinal pathology was reported in both clinical charts and radiology reports. Comparison of tabular- versus text-derived patient pathology was quantified with Cohen’s kappa values and total number of mentions, revealing limited agreement and more frequent reporting in tabular clinical charts.

| PATHOLOGY | KAPPA | TABULAR instances (n) | TEXT  instances (n) | Tabular/TEXT RATIO (%) |
| --- | --- | --- | --- | --- |
| Disc Pathology | 0.095 | 3310 | 1788 | 1.85 |
| Spinal Canal Stenosis | 0.12 | 3030 | 1188 | 2.55 |
| Facet Joint Arthropathy | 0.037 | 2941 | 330 | 8.91 |
| Sacroiliac Joint | 0.028 | 618 | 158 | 3.91 |
| Curvature | 0.23 | 870 | 320 | 2.72 |

## Supplemental Table 3: Finetuned Model Hyperparameters

Hyperparameter sets for finetuned models. The best model for each one-vs-one and one-vs-rest classification task and predictor datatype. The models were finetuned using a grid search and selected by the highest validation-set balanced accuracy. Bolded model variables correspond the model with the highest test-set balanced accuracy across all possible datatypes.

| **Labels (0 vs 1)** | **Datatype** | **Architecture** | **Finetuned Parameters** |
| --- | --- | --- | --- |
| **none_vs_nsaids** | **tabular_text** | XGBoost_best | {'model__alpha': 10, 'model__lambda': 100, 'model__learning_rate': 0.1, 'model__max_depth': 3, 'model__n_estimators': 100, 'model__objective': 'binary:logistic', 'model__scale_pos_weight': 1} |
| none_vs_nsaids | tabular | XGBoost_best | {'model__alpha': 0, 'model__lambda': 100, 'model__learning_rate': 0.5, 'model__max_depth': 3, 'model__n_estimators': 150, 'model__objective': 'binary:logistic', 'model__scale_pos_weight': 1} |
| none_vs_nsaids | text | XGBoost_best | {'model__alpha': 10, 'model__lambda': 10, 'model__learning_rate': 0.1, 'model__max_depth': 3, 'model__n_estimators': 50, 'model__objective': 'binary:logistic', 'model__scale_pos_weight': 1} |
| none_vs_nsaids | diagnoses_tabular | XGBoost_best | {'model__alpha': 10, 'model__lambda': 50, 'model__learning_rate': 0.01, 'model__max_depth': 6, 'model__n_estimators': 200, 'model__objective': 'binary:logistic', 'model__scale_pos_weight': 1} |
| none_vs_nsaids | diagnoses_text | XGBoost_best | {'model__alpha': 0, 'model__lambda': 10, 'model__learning_rate': 0.01, 'model__max_depth': 5, 'model__n_estimators': 150, 'model__objective': 'binary:logistic', 'model__scale_pos_weight': 1} |
| none_vs_nsaids | psychosocial_tabular | XGBoost_best | {'model__alpha': 10, 'model__lambda': 50, 'model__learning_rate': 0.1, 'model__max_depth': 3, 'model__n_estimators': 200, 'model__objective': 'binary:logistic', 'model__scale_pos_weight': 1} |
| **none_vs_opioids** | **tabular_text** | XGBoost_best | {'model__alpha': 10, 'model__lambda': 100, 'model__learning_rate': 0.01, 'model__max_depth': 6, 'model__n_estimators': 200, 'model__objective': 'binary:logistic', 'model__scale_pos_weight': 1.7} |
| none_vs_opioids | tabular | XGBoost_best | {'model__alpha': 10, 'model__lambda': 10, 'model__learning_rate': 0.01, 'model__max_depth': 4, 'model__n_estimators': 200, 'model__objective': 'binary:logistic', 'model__scale_pos_weight': 1.4} |
| none_vs_opioids | text | XGBoost_best | {'model__alpha': 50, 'model__lambda': 50, 'model__learning_rate': 0.1, 'model__max_depth': 6, 'model__n_estimators': 50, 'model__objective': 'binary:logistic', 'model__scale_pos_weight': 1.7} |
| none_vs_opioids | diagnoses_tabular | XGBoost_best | {'model__alpha': 10, 'model__lambda': 10, 'model__learning_rate': 0.1, 'model__max_depth': 3, 'model__n_estimators': 50, 'model__objective': 'binary:logistic', 'model__scale_pos_weight': 1} |
| none_vs_opioids | diagnoses_text | decision_tree_best | {'model__class_weight': 'balanced', 'model__criterion': 'gini', 'model__max_depth': 2, 'model__min_samples_leaf': 1, 'model__min_samples_split': 2, 'model__splitter': 'random'} |
| none_vs_opioids | psychosocial_tabular | XGBoost_best | {'model__alpha': 10, 'model__lambda': 100, 'model__learning_rate': 0.5, 'model__max_depth': 3, 'model__n_estimators': 50, 'model__objective': 'binary:logistic', 'model__scale_pos_weight': 1.4} |
| nsaids_vs_opioids | tabular_text | XGBoost_best | {'model__alpha': 10, 'model__lambda': 10, 'model__learning_rate': 0.1, 'model__max_depth': 3, 'model__n_estimators': 200, 'model__objective': 'binary:logistic', 'model__scale_pos_weight': 3.4} |
| **nsaids_vs_opioids** | **tabular** | XGBoost_best | {'model__alpha': 50, 'model__lambda': 10, 'model__learning_rate': 0.5, 'model__max_depth': 6, 'model__n_estimators': 50, 'model__objective': 'binary:logistic', 'model__scale_pos_weight': 1.4} |
| nsaids_vs_opioids | text | XGBoost_best | {'model__alpha': 0, 'model__lambda': 50, 'model__learning_rate': 0.01, 'model__max_depth': 6, 'model__n_estimators': 150, 'model__objective': 'binary:logistic', 'model__scale_pos_weight': 1.4} |
| nsaids_vs_opioids | diagnoses_tabular | XGBoost_best | {'model__alpha': 0, 'model__lambda': 10, 'model__learning_rate': 0.01, 'model__max_depth': 4, 'model__n_estimators': 200, 'model__objective': 'binary:logistic', 'model__scale_pos_weight': 1} |
| nsaids_vs_opioids | diagnoses_text | decision_tree_best | {'model__class_weight': 'balanced', 'model__criterion': 'entropy', 'model__max_depth': 8, 'model__min_samples_leaf': 4, 'model__min_samples_split': 10, 'model__splitter': 'best'} |
| nsaids_vs_opioids | psychosocial_tabular | XGBoost_best | {'model__alpha': 10, 'model__lambda': 10, 'model__learning_rate': 0.1, 'model__max_depth': 4, 'model__n_estimators': 100, 'model__objective': 'binary:logistic', 'model__scale_pos_weight': 1.4} |
| rest_vs_none | tabular_text | random_forest_best | {'model__bootstrap': False, 'model__class_weight': 'balanced', 'model__max_depth': 10, 'model__min_samples_leaf': 1, 'model__min_samples_split': 2, 'model__n_estimators': 50} |
| **rest_vs_none** | **tabular** | XGBoost_best | {'model__alpha': 0, 'model__lambda': 0, 'model__learning_rate': 0.1, 'model__max_depth': 3, 'model__n_estimators': 50, 'model__objective': 'binary:logistic', 'model__scale_pos_weight': 1} |
| rest_vs_none | text | XGBoost_best | {'model__alpha': 10, 'model__lambda': 10, 'model__learning_rate': 0.1, 'model__max_depth': 3, 'model__n_estimators': 50, 'model__objective': 'binary:logistic', 'model__scale_pos_weight': 1} |
| rest_vs_none | diagnoses_tabular | XGBoost_best | {'model__alpha': 10, 'model__lambda': 100, 'model__learning_rate': 0.1, 'model__max_depth': 4, 'model__n_estimators': 150, 'model__objective': 'binary:logistic', 'model__scale_pos_weight': 1} |
| rest_vs_none | diagnoses_text | XGBoost_best | {'model__alpha': 0, 'model__lambda': 50, 'model__learning_rate': 0.01, 'model__max_depth': 6, 'model__n_estimators': 150, 'model__objective': 'binary:logistic', 'model__scale_pos_weight': 1} |
| rest_vs_none | psychosocial_tabular | XGBoost_best | {'model__alpha': 10, 'model__lambda': 0, 'model__learning_rate': 0.1, 'model__max_depth': 3, 'model__n_estimators': 50, 'model__objective': 'binary:logistic', 'model__scale_pos_weight': 1} |
| **rest_vs_nsaids** | **tabular_text** | XGBoost_best | {'model__alpha': 10, 'model__lambda': 50, 'model__learning_rate': 0.01, 'model__max_depth': 3, 'model__n_estimators': 150, 'model__objective': 'binary:logistic', 'model__scale_pos_weight': 1} |
| rest_vs_nsaids | tabular | XGBoost_best | {'model__alpha': 10, 'model__lambda': 50, 'model__learning_rate': 0.01, 'model__max_depth': 4, 'model__n_estimators': 150, 'model__objective': 'binary:logistic', 'model__scale_pos_weight': 1} |
| rest_vs_nsaids | text | XGBoost_best | {'model__alpha': 0, 'model__lambda': 100, 'model__learning_rate': 0.1, 'model__max_depth': 4, 'model__n_estimators': 150, 'model__objective': 'binary:logistic', 'model__scale_pos_weight': 1.7} |
| rest_vs_nsaids | diagnoses_tabular | XGBoost_best | {'model__alpha': 10, 'model__lambda': 10, 'model__learning_rate': 0.01, 'model__max_depth': 4, 'model__n_estimators': 150, 'model__objective': 'binary:logistic', 'model__scale_pos_weight': 1} |
| rest_vs_nsaids | diagnoses_text | decision_tree_best | {'model__class_weight': 'balanced', 'model__criterion': 'gini', 'model__max_depth': 2, 'model__min_samples_leaf': 1, 'model__min_samples_split': 2, 'model__splitter': 'random'} |
| rest_vs_nsaids | psychosocial_tabular | XGBoost_best | {'model__alpha': 10, 'model__lambda': 50, 'model__learning_rate': 1.0, 'model__max_depth': 6, 'model__n_estimators': 50, 'model__objective': 'binary:logistic', 'model__scale_pos_weight': 1} |
| rest_vs_opioids | tabular_text | XGBoost_best | {'model__alpha': 50, 'model__lambda': 50, 'model__learning_rate': 0.5, 'model__max_depth': 4, 'model__n_estimators': 50, 'model__objective': 'binary:logistic', 'model__scale_pos_weight': 4.8} |
| **rest_vs_opioids** | **tabular** | XGBoost_best | {'model__alpha': 100, 'model__lambda': 50, 'model__learning_rate': 0.5, 'model__max_depth': 6, 'model__n_estimators': 50, 'model__objective': 'binary:logistic', 'model__scale_pos_weight': 1.7} |
| rest_vs_opioids | text | XGBoost_best | {'model__alpha': 10, 'model__lambda': 50, 'model__learning_rate': 0.1, 'model__max_depth': 4, 'model__n_estimators': 150, 'model__objective': 'binary:logistic', 'model__scale_pos_weight': 8.2} |
| rest_vs_opioids | diagnoses_tabular | XGBoost_best | {'model__alpha': 50, 'model__lambda': 50, 'model__learning_rate': 0.01, 'model__max_depth': 3, 'model__n_estimators': 150, 'model__objective': 'binary:logistic', 'model__scale_pos_weight': 1} |
| rest_vs_opioids | diagnoses_text | decision_tree_best | {'model__class_weight': 'balanced', 'model__criterion': 'gini', 'model__max_depth': 8, 'model__min_samples_leaf': 2, 'model__min_samples_split': 10, 'model__splitter': 'random'} |
| rest_vs_opioids | psychosocial_tabular | XGBoost_best | {'model__alpha': 50, 'model__lambda': 50, 'model__learning_rate': 0.1, 'model__max_depth': 4, 'model__n_estimators': 100, 'model__objective': 'binary:logistic', 'model__scale_pos_weight': 1.7} |

## Supplemental Table 4: Finetuned Model Test-Set Performance Metrics

Test-set performance metrics for finetuned models across six outcome pairs and six patient profile compositions. One-vs-one and one-vs-rest classification outcomes assess relative differences in medication prescription (*none_vs_nsaids, none_vs_opioids, nsaids_vs_opioids)* and the likelihood of prescribing a specific class (*rest_vs_none, rest_vs_nsaids, rest_vs_opioids*). Patient profile compositions were defined based on documentation sources and biopsychosocial features, as specified in ***Figure 1 (e)***. Performance metrics include precision, recall, sensitivity, F1-score, balanced accuracy, and AUC, with mean values and 95% confidence interval estimated from bootstrapping (n=1,000). Bolded model variables indicate the highest balanced accuracy for the given label, while text color indicates performance bins (high: [0.5,1], medium: [0.55, 0.6), low: [0,0.55)) for balanced accuracy and AUC. Medication prescription classification proved challenging, with mean balanced accuracies ranging from 0.49 to 0.67. Opioid-related classifications (*none_vs_opioid, nsaid_vs_opioid, rest_vs_opioid*) exhibited lower performance metrics suggesting that prescribing patterns may be primarily influenced by factors not capture through structured EMR documentation, such as detailed accounts of patient-reported pain, which were beyond the scope of this study. Nonetheless, these models can still serve as weak predictors to explore associations in past opioid prescriptions.

| **Labels (0 vs 1)** | **Datatype** | **Precision** | **Recall** | **Sensitivity** | **F1-Score** | **Balanced Acc** | **AUC** |
| --- | --- | --- | --- | --- | --- | --- | --- |
| **none_vs_nsaids** | **tabular_text** | 0.61 [0.55, 0.67] | 0.60 [0.54, 0.66] | 0.71 [0.66, 0.76] | 0.61 [0.55, 0.66] | 0.66 [0.62, 0.70] | 0.71 [0.67, 0.75] |
| none_vs_nsaids | tabular | 0.57 [0.50, 0.64] | 0.54 [0.48, 0.61] | 0.70 [0.64, 0.75] | 0.56 [0.50, 0.61] | 0.62 [0.58, 0.66] | 0.67 [0.63, 0.72] |
| none_vs_nsaids | text | 0.54 [0.47, 0.61] | 0.46 [0.39, 0.52] | 0.71 [0.66, 0.76] | 0.50 [0.44, 0.55] | 0.59 [0.54, 0.63] | 0.64 [0.59, 0.68] |
| none_vs_nsaids | diagnoses_tabular | 0.55 [0.49, 0.61] | 0.58 [0.51, 0.64] | 0.64 [0.59, 0.69] | 0.56 [0.51, 0.61] | 0.61 [0.57, 0.65] | 0.66 [0.61, 0.71] |
| none_vs_nsaids | diagnoses_text | 0.51 [0.45, 0.56] | 0.58 [0.52, 0.64] | 0.57 [0.51, 0.63] | 0.54 [0.49, 0.59] | 0.58 [0.54, 0.62] | 0.60 [0.55, 0.65] |
| none_vs_nsaids | psychosocial_tabular | 0.51 [0.45, 0.57] | 0.53 [0.46, 0.59] | 0.62 [0.56, 0.67] | 0.52 [0.46, 0.57] | 0.57 [0.53, 0.61] | 0.60 [0.55, 0.65] |
| **none_vs_opioids** | **tabular_text** | 0.29 [0.22, 0.36] | 0.75 [0.64, 0.85] | 0.60 [0.55, 0.65] | 0.42 [0.34, 0.50] | 0.68 [0.62, 0.74] | 0.70 [0.63, 0.77] |
| none_vs_opioids | tabular | 0.28 [0.21, 0.35] | 0.69 [0.58, 0.79] | 0.61 [0.55, 0.67] | 0.40 [0.32, 0.48] | 0.65 [0.59, 0.71] | 0.71 [0.64, 0.78] |
| none_vs_opioids | text | 0.27 [0.20, 0.35] | 0.59 [0.47, 0.70] | 0.66 [0.61, 0.71] | 0.37 [0.29, 0.45] | 0.62 [0.56, 0.69] | 0.65 [0.58, 0.71] |
| none_vs_opioids | diagnoses_tabular | 0.30 [0.22, 0.39] | 0.50 [0.38, 0.62] | 0.75 [0.70, 0.79] | 0.37 [0.29, 0.46] | 0.62 [0.56, 0.69] | 0.70 [0.62, 0.77] |
| none_vs_opioids | diagnoses_text | 0.45 [0.12, 0.81] | 0.06 [0.01, 0.13] | 0.98 [0.97, 1.00] | 0.10 [0.03, 0.22] | 0.52 [0.50, 0.56] | 0.63 [0.56, 0.71] |
| none_vs_opioids | psychosocial_tabular | 0.25 [0.18, 0.33] | 0.48 [0.36, 0.59] | 0.70 [0.64, 0.75] | 0.33 [0.25, 0.41] | 0.59 [0.52, 0.65] | 0.60 [0.53, 0.67] |
| nsaids_vs_opioids | tabular_text | 0.25 [0.19, 0.32] | 0.60 [0.49, 0.71] | 0.47 [0.41, 0.53] | 0.35 [0.27, 0.43] | 0.54 [0.47, 0.60] | 0.59 [0.51, 0.66] |
| **nsaids_vs_opioids** | **tabular** | 0.28 [0.21, 0.35] | 0.65 [0.53, 0.75] | 0.52 [0.46, 0.58] | 0.39 [0.31, 0.47] | 0.58 [0.52, 0.65] | 0.61 [0.53, 0.69] |
| nsaids_vs_opioids | text | 0.27 [0.20, 0.34] | 0.69 [0.58, 0.80] | 0.45 [0.39, 0.51] | 0.39 [0.30, 0.46] | 0.57 [0.51, 0.63] | 0.57 [0.50, 0.65] |
| nsaids_vs_opioids | diagnoses_tabular | 0.27 [0.19, 0.35] | 0.48 [0.37, 0.60] | 0.62 [0.56, 0.68] | 0.35 [0.26, 0.43] | 0.55 [0.48, 0.62] | 0.60 [0.53, 0.67] |
| nsaids_vs_opioids | diagnoses_text | 0.27 [0.14, 0.40] | 0.19 [0.10, 0.29] | 0.85 [0.80, 0.89] | 0.22 [0.12, 0.32] | 0.52 [0.47, 0.57] | 0.54 [0.46, 0.62] |
| nsaids_vs_opioids | psychosocial_tabular | 0.28 [0.18, 0.38] | 0.34 [0.23, 0.46] | 0.74 [0.69, 0.80] | 0.31 [0.21, 0.40] | 0.54 [0.48, 0.61] | 0.53 [0.45, 0.61] |
| rest_vs_none | tabular_text | 0.58 [0.53, 0.63] | 0.60 [0.54, 0.66] | 0.55 [0.50, 0.60] | 0.59 [0.54, 0.63] | 0.58 [0.54, 0.61] | 0.62 [0.58, 0.66] |
| **rest_vs_none** | **tabular** | 0.64 [0.59, 0.69] | 0.67 [0.62, 0.72] | 0.61 [0.56, 0.66] | 0.65 [0.61, 0.70] | 0.64 [0.60, 0.68] | 0.69 [0.65, 0.73] |
| rest_vs_none | text | 0.58 [0.53, 0.63] | 0.68 [0.62, 0.72] | 0.50 [0.44, 0.55] | 0.62 [0.58, 0.67] | 0.59 [0.55, 0.62] | 0.64 [0.59, 0.68] |
| rest_vs_none | diagnoses_tabular | 0.63 [0.58, 0.68] | 0.63 [0.57, 0.68] | 0.62 [0.57, 0.68] | 0.63 [0.58, 0.67] | 0.62 [0.58, 0.66] | 0.66 [0.62, 0.71] |
| rest_vs_none | diagnoses_text | 0.59 [0.54, 0.65] | 0.63 [0.57, 0.68] | 0.56 [0.50, 0.61] | 0.61 [0.57, 0.66] | 0.59 [0.56, 0.63] | 0.62 [0.58, 0.66] |
| rest_vs_none | psychosocial_tabular | 0.57 [0.51, 0.62] | 0.62 [0.57, 0.68] | 0.51 [0.45, 0.57] | 0.59 [0.55, 0.64] | 0.57 [0.53, 0.60] | 0.61 [0.57, 0.66] |
| **rest_vs_nsaids** | **tabular_text** | 0.50 [0.45, 0.56] | 0.59 [0.52, 0.65] | 0.64 [0.59, 0.69] | 0.54 [0.49, 0.60] | 0.61 [0.57, 0.65] | 0.64 [0.60, 0.69] |
| rest_vs_nsaids | tabular | 0.49 [0.43, 0.56] | 0.55 [0.48, 0.61] | 0.66 [0.61, 0.70] | 0.52 [0.46, 0.57] | 0.60 [0.56, 0.64] | 0.65 [0.60, 0.69] |
| rest_vs_nsaids | text | 0.42 [0.37, 0.48] | 0.71 [0.65, 0.77] | 0.41 [0.36, 0.46] | 0.53 [0.48, 0.58] | 0.56 [0.52, 0.60] | 0.58 [0.53, 0.63] |
| rest_vs_nsaids | diagnoses_tabular | 0.49 [0.43, 0.55] | 0.56 [0.50, 0.62] | 0.63 [0.58, 0.68] | 0.52 [0.47, 0.57] | 0.60 [0.56, 0.64] | 0.64 [0.59, 0.68] |
| rest_vs_nsaids | diagnoses_text | 0.42 [0.32, 0.51] | 0.18 [0.13, 0.23] | 0.85 [0.82, 0.88] | 0.25 [0.19, 0.31] | 0.51 [0.49, 0.54] | 0.54 [0.49, 0.58] |
| rest_vs_nsaids | psychosocial_tabular | 0.45 [0.38, 0.51] | 0.41 [0.35, 0.47] | 0.69 [0.64, 0.74] | 0.43 [0.37, 0.48] | 0.55 [0.51, 0.59] | 0.55 [0.51, 0.60] |
| rest_vs_opioids | tabular_text | 0.14 [0.10, 0.19] | 0.47 [0.35, 0.59] | 0.65 [0.61, 0.69] | 0.22 [0.16, 0.28] | 0.56 [0.50, 0.62] | 0.61 [0.53, 0.68] |
| **rest_vs_opioids** | **tabular** | 0.17 [0.12, 0.22] | 0.53 [0.40, 0.65] | 0.68 [0.64, 0.72] | 0.26 [0.18, 0.32] | 0.60 [0.54, 0.66] | 0.63 [0.56, 0.70] |
| rest_vs_opioids | text | 0.13 [0.10, 0.17] | 0.72 [0.61, 0.83] | 0.41 [0.37, 0.45] | 0.22 [0.17, 0.28] | 0.56 [0.51, 0.62] | 0.56 [0.49, 0.63] |
| rest_vs_opioids | diagnoses_tabular | 0.13 [0.09, 0.16] | 0.59 [0.47, 0.71] | 0.50 [0.46, 0.54] | 0.21 [0.15, 0.26] | 0.54 [0.48, 0.61] | 0.58 [0.51, 0.65] |
| rest_vs_opioids | diagnoses_text | 0.17 [0.04, 0.35] | 0.06 [0.01, 0.12] | 0.97 [0.95, 0.98] | 0.09 [0.02, 0.18] | 0.51 [0.49, 0.54] | 0.55 [0.47, 0.62] |
| rest_vs_opioids | psychosocial_tabular | 0.12 [0.09, 0.17] | 0.47 [0.36, 0.59] | 0.59 [0.55, 0.63] | 0.20 [0.14, 0.25] | 0.53 [0.47, 0.60] | 0.55 [0.48, 0.62] |

## Supplemental Figure 1: SHAP Feature Importance Bar Plot

SHAP (SHapley Additive exPlanations) values quantify the relative importance of patient profile features for each one-vs-one (OVO) and one-vs-rest (OVR) medication prediction task. Global feature importance ranking, calculated from the average magnitude of SHAP values across patients, revealed influential clinical predictors of pharmacological intervention decisions. *Year_at_first_imaging* (top 7), *disc_pathology* (top 7), *stenosis* (top 8), and *race_ethnicity* (top 8), consistently emerge as key predictors across medication outcomes, while *LBP_duration*, *LBP_laterality*, *negative_psych_state*, and *primary_insurance* exhibit variable importance.


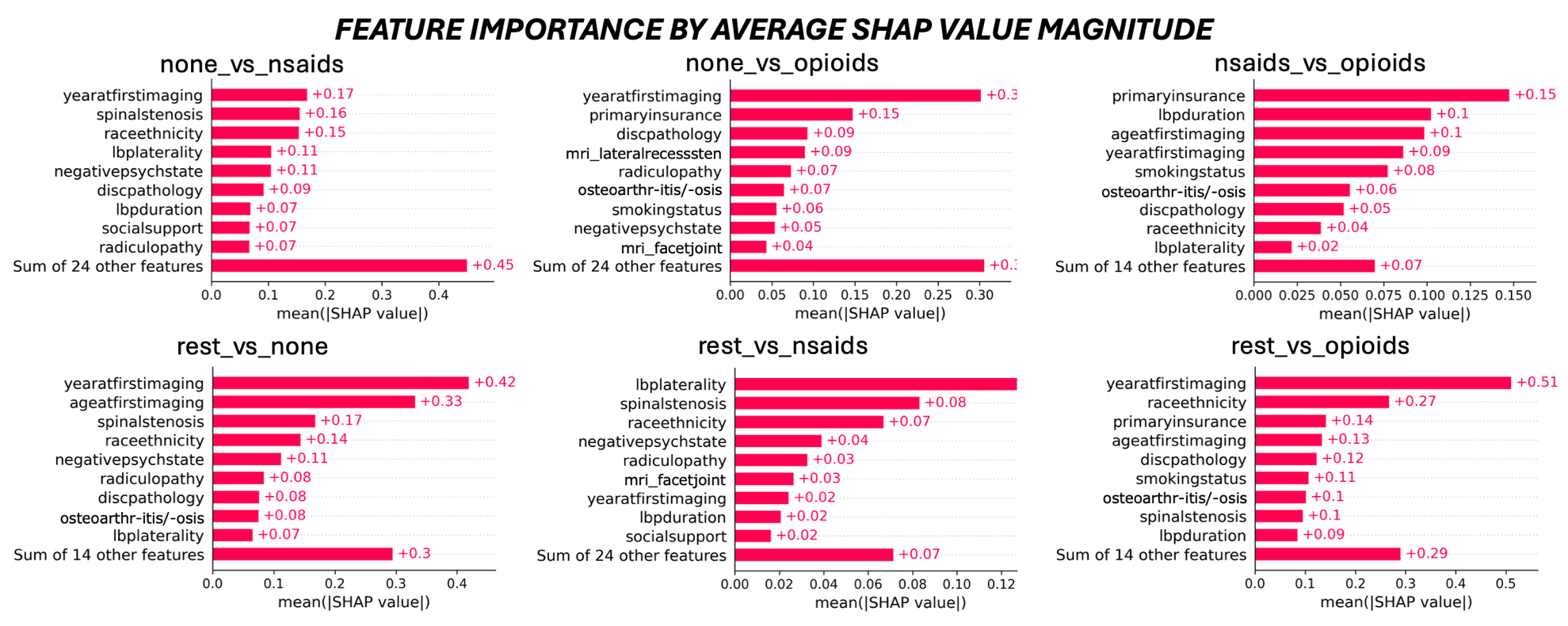


## Supplementary Table 5: Patient Profiles (clinical charts)

Lower back pain patient profiles (n=4,077) from EMR clinical charts include demographic, psychosocial, and diagnostic features as numerical (continuous values), categorical (multiple values), or binary (one value). Descriptive statistics report the count and percentage prevalence of each value overall and stratified by medication outcome. Statistical comparisons across medication groups were conducted using chi-squared tests with Bonferroni correction for categorical and binary features, and one-way ANOVA for numerical features, with p-values < 0.05 indicating statistical significance. P-values < 0.05 indicated statistical significance in NSAID and opioid prescriptions among demographics (age at first imaging, year at first imaging, and obesity), psychosocial determinants (race/ethnicity, smoking status, limited social support, primary insurance, and a history of anxiety or depression), and medical chart diagnoses (LBP duration, disc pathology, spinal stenosis, facet joint arthropathy, sacroiliac joint, sciatica, and diabetes).

| *Grouped by Intervention*  *FEATURE* | *VALUE* | Overall  N (%) or  MEAN (SD) | UNSPECIFIED N (%) or  MEAN (SD) | NSAID  N (%) or  MEAN (SD) | Opioid  N (%) or  MEAN (SD) | P-Value |
| --- | --- | --- | --- | --- | --- | --- |
| **---** | **---** | 4077 | 2115 | 1519 | 443 | **---** |
| **--- *DEMOGRAPHICS* ---** | **---** | **---** | **---** | **---** | **---** | **---** |
| **AGE AT FIRST IMAGING** | **---** | 48.5 (16.9) | 47.5 (17.1) | 49.8 (16.7) | 49.3 (15.5) | <0.001 |
| **YEAR AT FIRST IMAGING** | **---** | 2018.7 (3.2) | 2018.6 (3.4) | 2019.0 (3.0) | 2018.3 (3.1) | <0.001 |
| **SEX** | **MALE (1)** | 2250 (55.2) | 811 (53.4) | 238 (53.7) | 1201 (56.8) | 0.103 |
|  | **FEMALE (0)** | 1827 (44.8) | 914 (43.2) | 708 (46.6) | 205 (46.3) | **---** |
| **OBESITY** | **NOT OBESE** | 3862 (94.7) | 2044 (96.6) | 1414 (93.1) | 404 (91.2) | <0.001 |
|  | **OBESE** | 215 (5.3) | 105 (6.9) | 39 (8.8) | 71 (3.4) | **---** |
| **--- *PSYCHOSOCIAL* ---** | **---** | **---** | **---** | **---** | **---** | **---** |
| **PREFERRED LANGUAGE** | **ENGLISH** | 3826 (93.8) | 1417 (93.3) | 413 (93.2) | 1996 (94.4) | 0.343 |
|  | **NOT ENGLISH** | 251 (6.2) | 119 (5.6) | 102 (6.7) | 30 (6.8) | **---** |
| **RACE ETHNICITY** | **WHITE** | 2327 (57.1) | 886 (58.3) | 260 (58.7) | 1181 (55.8) | <0.001 |
|  | **ASIAN** | 584 (14.3) | 278 (13.1) | 256 (16.9) | 50 (11.3) | **---** |
|  | **OTHER** | 575 (14.1) | 197 (13.0) | 50 (11.3) | 328 (15.5) | **---** |
|  | **LATINX** | 396 (9.7) | 112 (7.4) | 59 (13.3) | 225 (10.6) | **---** |
|  | **BLACK** | 195 (4.8) | 68 (4.5) | 24 (5.4) | 103 (4.9) | **---** |
| **SMOKING STATUS** | **NEVER** | 3133 (76.8) | 1151 (75.8) | 311 (70.2) | 1671 (79.0) | <0.001 |
|  | **FORMER** | 725 (17.8) | 339 (16.0) | 292 (19.2) | 94 (21.2) | **---** |
|  | **SMOKER** | 219 (5.4) | 76 (5.0) | 38 (8.6) | 105 (5.0) | **---** |
| **SOCIAL SUPPORT** | **PARTNERED** | 2296 (56.3) | 1135 (53.7) | 911 (60.0) | 250 (56.4) | 0.001 |
|  | **SINGLE/ SEPARATED** | 1781 (43.7) | 608 (40.0) | 193 (43.6) | 980 (46.3) | **---** |
| **PRIMARY INSURANCE** | **PPO** | 2220 (54.5) | 794 (52.3) | 216 (48.8) | 1210 (57.2) | <0.001 |
|  | **MEDICARE** | 731 (17.9) | 319 (21.0) | 76 (17.2) | 336 (15.9) | **---** |
|  | **HMP/POS/EPO** | 533 (13.1) | 256 (12.1) | 210 (13.8) | 67 (15.1) | **---** |
|  | **MEDICAID** | 492 (12.1) | 175 (11.5) | 68 (15.3) | 249 (11.8) | **---** |
|  | **OTHER** | 101 (2.5) | 21 (1.4) | 16 (3.6) | 64 (3.0) | **---** |
| **NEGATIVE PSYCH STATE** | **NOT NEGATIVE** | 3621 (88.8) | 1983 (93.8) | 1274 (83.9) | 364 (82.2) | <0.001 |
| **(ANXIETY OR DEPRESSION)** | **NEGATIVE** | 456 (11.2) | 245 (16.1) | 79 (17.8) | 132 (6.2) | **---** |
| **--- *DIAGNOSES (CHARTS)* ---** | **---** | **---** | **---** | **---** | **---** | **---** |
| **LBP DURATION** | **UNSPECIFIED** | 2873 (70.5) | 1631 (77.1) | 946 (62.3) | 296 (66.8) | <0.001 |
|  | **CHRONIC** | 968 (23.7) | 479 (31.5) | 94 (21.2) | 395 (18.7) | **---** |
|  | **ACUTE** | 236 (5.8) | 94 (6.2) | 53 (12.0) | 89 (4.2) | **---** |
| **LBP LATERALITY** | **UNSPECIFIED** | 3021 (74.1) | 1702 (80.5) | 999 (65.8) | 320 (72.2) | <0.001 |
| **LBP LATERALITY** | **UNILATERAL** | 589 (14.4) | 212 (10.0) | 295 (19.4) | 82 (18.5) | **---** |
| **LBP LATERALITY** | **BILATERAL** | 467 (11.5) | 201 (9.5) | 225 (14.8) | 41 (9.3) | **---** |
| **NUMBNESS / TINGLING** | **TRUE** | 289 (7.1) | 136 (6.4) | 123 (8.1) | 30 (6.8) | 0.149 |
| **RADICULOPATHY** | **TRUE** | 1856 (45.5) | 830 (39.2) | 802 (52.8) | 224 (50.6) | <0.001 |
| **DISC PATHOLOGY** | **TRUE** | 1788 (43.9) | 718 (47.3) | 242 (54.6) | 828 (39.1) | <0.001 |
| **SPINAL STENOSIS** | **TRUE** | 1188 (29.1) | 551 (36.3) | 169 (38.1) | 468 (22.1) | <0.001 |
| **FACET JOINT ARTHROPATHY** | **TRUE** | 330 (8.1) | 167 (11.0) | 36 (8.1) | 127 (6.0) | <0.001 |
| **SACROILIAC JOINT** | **TRUE** | 158 (3.9) | 79 (5.2) | 10 (2.3) | 69 (3.3) | 0.002 |
| **SCOLIOSIS** | **TRUE** | 320 (7.8) | 126 (8.3) | 37 (8.4) | 157 (7.4) | 0.576 |
| **SCIATICA** | **TRUE** | 1193 (29.3) | 553 (36.4) | 156 (35.2) | 484 (22.9) | <0.001 |
| **OSTEO -PENIA / -POROSIS** | **TRUE** | 103 (2.5) | 30 (1.4) | 58 (3.8) | 15 (3.4) | <0.001 |
| **OSTEOARTHR - ITIS / - OSIS** | **TRUE** | 486 (11.9) | 161 (7.6) | 237 (15.6) | 88 (19.9) | <0.001 |
| **FIBROMYALGIA / FIBROSIS** | **TRUE** | 89 (2.2) | 43 (2.8) | 9 (2.0) | 37 (1.7) | 0.087 |

Supplementary Table 6: Demographic Missingness

Demographic patient feature distributions prior to missing data imputation, showing counts and percentages for each categorical value both overall and stratified by medication outcome. Chi-squared tests with Bonferroni correction for multiple comparisons were performed to assess differences in value distributions across medication groups. Missing data rates varied from 6.4% (race / ethnicity) to 32.0% (primary insurance) and all demographic features demonstrated statistically significant differences across medication outcomes (p < 0.05).

| *Grouped by Intervention*  feature | VALUE | Overall  N (%) | UNSPECIFIED N (%) | NSAID  N (%) | Opioid  N (%) | P-Value |
| --- | --- | --- | --- | --- | --- | --- |
| **---** | ***---*** | 4077 | 2115 | 1519 | 443 | **---** |
| **RACEETHNICITY** | **WHITE** | 2240 (54.9) | 1119 (52.9) | 867 (57.1) | 254 (57.3) | <0.001 |
|  | **ASIAN** | 583 (14.3) | 277 (13.1) | 256 (16.9) | 50 (11.3) | **---** |
|  | **OTHER** | 456 (11.2) | 250 (11.8) | 166 (10.9) | 40 (9.0) | **---** |
|  | **LATINX** | 357 (8.8) | 193 (9.1) | 108 (7.1) | 56 (12.6) | **---** |
|  | **BLACK** | 178 (4.4) | 93 (4.4) | 63 (4.1) | 22 (5.0) | **---** |
|  | **UNKNOWN** | 263 (6.4) | 183 (8.7) | 59 (3.9) | 21 (4.7) | **---** |
| **SMOKINGSTATUS** | **SMOKER** | 213 (5.2) | 100 (4.7) | 76 (5.0) | 37 (8.4) | <0.001 |
|  | **FORMER** | 687 (16.9) | 307 (14.5) | 288 (19.0) | 92 (20.8) | **---** |
|  | **NEVER** | 2661 (65.3) | 1279 (60.5) | 1101 (72.5) | 281 (63.4) | **---** |
|  | **UNKNOWN** | 516 (12.6) | 429 (20.3) | 54 (3.6) | 33 (7.4) | **---** |
| **SOCIALSUPPORT** | **PARTNERED** | 2082 (51.1) | 980 (46.3) | 864 (56.9) | 238 (53.7) | <0.001 |
|  | **SINGLE/ SEPARATED** | 1658 (40.7) | 895 (42.3) | 573 (37.7) | 190 (42.9) | **---** |
|  | **UNKNOWN** | 337 (8.2) | 240 (11.3) | 82 (5.4) | 15 (3.4) | **---** |
| **PRIMARYINSURANCE** | **PPO** | 1412 (34.6) | 660 (31.2) | 586 (38.6) | 166 (37.5) | <0.001 |
|  | **MEDICARE** | 546 (13.4) | 231 (10.9) | 253 (16.7) | 62 (14.0) | **---** |
|  | **MEDICAID** | 263 (6.5) | 95 (4.5) | 113 (7.4) | 55 (12.4) | **---** |
|  | **HMP/POS/EPO** | 454 (11.1) | 206 (9.7) | 187 (12.3) | 61 (13.8) | **---** |
|  | **OTHER** | 98 (2.4) | 62 (2.9) | 21 (1.4) | 15 (3.4) | **---** |
|  | **UNKNOWN** | 1304 (32.0) | 861 (40.7) | 359 (23.6) | 84 (19.0) | **---** |

Supplementary Table 7: Assessment of the Type of Missingness

Statistical testing determined the appropriate imputation strategy for demographic variables through evaluation of missingness patterns (*y_miss*) using both Little's missing completely at random (MCAR) test and logistic regression-based missing at random (MAR) assessment. For each demographic feature, the distribution of values within reported and missing data subsets are specified by counts and percentages. Significant associations between predictor variables and missingness patterns (p < 0.001) rejected the MCAR hypothesis. Missingness patterns revealed specific dependencies: *race_ethnicity* missingness correlated with *age_at_first_imaging*; *social_support* missingness corrleated with *race_ethnicity*; *smoking_status* missingness correlated with *social_support* and *primary_insurance*; and *primary_insurance* missingness correlated with *year_at_first_imaging*. Logistic regression models predicting missingness achieved balanced accuracies of 0.5, indicating the data were not MAR.

| *Grouped by RACE/ETHNICITY*  FEATURE VALUE | | Overall  N (%) or  MEAN (SD) | REPORTED  N (%) or  MEAN (SD) | MISSING  N (%) or  MEAN (SD) | P-VALUE |
| --- | --- | --- | --- | --- | --- |
| --- | **---** | 2440 | 2363 | 77 | **---** |
| AGE AT FIRST IMAGING | **---** | 49.3 (16.6) | 49.6 (16.6) | 41.1 (15.1) | **<0.001** |
| YEAR AT FIRST IMAGING | **---** | 2018.9 (3.2) | 2018.9 (3.2) | 2018.1 (3.7) | 0.067 |
| SEX | **MALE** | 1309 (53.6) | 1269 (53.7) | 40 (51.9) | 0.851 |
|  | **FEMALE** | 1131 (46.4) | 1094 (46.3) | 37 (48.1) | **---** |
| PREFERRED LANGUAGE | **ENGLISH** | 2296 (94.1) | 2220 (93.9) | 76 (98.7) | 0.086 |
|  | **NON-ENGLISH** | 144 (5.9) | 143 (6.1) | 1 (1.3) | **---** |
| SOCIAL SUPPORT | **SINGLE/SEPARATED** | 1033 (42.3) | 989 (41.9) | 44 (57.1) | 0.011 |
|  | **PARTNERED** | 1407 (57.7) | 1374 (58.1) | 33 (42.9) | **---** |
| PRIMARY INSURANCE | **PPO** | 1264 (51.8) | 1214 (51.4) | 50 (64.9) | 0.051 |
|  | **MEDICARE** | 500 (20.5) | 493 (20.9) | 7 (9.1) | **---** |
|  | **HMO/POS/EPO** | 402 (16.5) | 389 (16.5) | 13 (16.9) | **---** |
|  | **MEDICAID** | 221 (9.1) | 214 (9.1) | 7 (9.1) | **---** |
|  | **OTHER** | 53 (2.2) | 53 (2.2) |  | **---** |
| SMOKING STATUS | **NEVER** | 1823 (74.7) | 1759 (74.4) | 64 (83.1) | 0.126 |
|  | **FORMER** | 474 (19.4) | 466 (19.7) | 8 (10.4) | **---** |
|  | **SMOKER** | 143 (5.9) | 138 (5.8) | 5 (6.5) | **---** |
| *Grouped by SOCIAL SUPPORT* | | Overall  N (%) or  MEAN (SD) | REPORTED  N (%) or  MEAN (SD) | MISSING  N (%) or  MEAN (SD) | P-VALUE |
| --- | **---** | 2461 | 2363 | 98 | **---** |
| AGE AT FIRST IMAGING | **---** | 49.5 (16.6) | 49.6 (16.6) | 47.7 (15.2) | 0.239 |
| YEAR AT FIRST IMAGING | **---** | 2018.9 (3.2) | 2018.9 (3.2) | 2019.4 (2.8) | 0.061 |
| SEX | **MALE** | 1322 (53.7) | 1269 (53.7) | 53 (54.1) | 1 |
|  | **FEMALE** | 1139 (46.3) | 1094 (46.3) | 45 (45.9) | **---** |
| PREFERRED LANGUAGE | **ENGLISH** | 2306 (93.7) | 2220 (93.9) | 86 (87.8) | 0.024 |
|  | **NON-ENGLISH** | 155 (6.3) | 143 (6.1) | 12 (12.2) | **---** |
| RACE/ETHNICITY | **WHITE** | 1438 (58.4) | 1400 (59.2) | 38 (38.8) | **<0.001** |
|  | **ASIAN** | 397 (16.1) | 380 (16.1) | 17 (17.3) | **---** |
|  | **OTHER** | 276 (11.2) | 246 (10.4) | 30 (30.6) | **---** |
|  | **LATINX** | 232 (9.4) | 226 (9.6) | 6 (6.1) | **---** |
|  | **BLACK OR AFRICAN AMERICAN** | 118 (4.8) | 111 (4.7) | 7 (7.1) | **---** |
| PRIMARY INSURANCE | **PPO** | 1263 (51.3) | 1214 (51.4) | 49 (50.0) | 0.222 |
|  | **MEDICARE** | 507 (20.6) | 493 (20.9) | 14 (14.3) | **---** |
|  | **HMO/POS/EPO** | 411 (16.7) | 389 (16.5) | 22 (22.4) | **---** |
|  | **MEDICAID** | 226 (9.2) | 214 (9.1) | 12 (12.2) | **---** |
|  | **OTHER** | 54 (2.2) | 53 (2.2) | 1 (1.0) | **---** |
| SMOKING STATUS | **NEVER** | 1836 (74.6) | 1759 (74.4) | 77 (78.6) | 0.385 |
|  | **FORMER** | 480 (19.5) | 466 (19.7) | 14 (14.3) | **---** |
|  | **SMOKER** | 145 (5.9) | 138 (5.8) | 7 (7.1) | **---** |
| *Grouped by SMOKING STATUS* | | Overall  N (%) or  MEAN (SD) | REPORTED  N (%) or  MEAN (SD) | MISSING  N (%) or  MEAN (SD) | P-VALUE |
| --- | **---** | 2520 | 2363 | 157 | **---** |
| AGE AT FIRST IMAGING | **---** | 49.5 (16.6) | 49.6 (16.6) | 47.7 (16.1) | 0.159 |
| YEAR AT FIRST IMAGING | **---** | 2018.9 (3.2) | 2018.9 (3.2) | 2018.3 (4.0) | 0.061 |
| SEX | **MALE** | 1358 (53.9) | 1269 (53.7) | 89 (56.7) | 0.52 |
|  | **FEMALE** | 1162 (46.1) | 1094 (46.3) | 68 (43.3) | **---** |
| PREFERRED LANGUAGE | **ENGLISH** | 2359 (93.6) | 2220 (93.9) | 139 (88.5) | 0.012 |
|  | **NON-ENGLISH** | 161 (6.4) | 143 (6.1) | 18 (11.5) | **---** |
| RACE/ETHNICITY | **WHITE** | 1488 (59.0) | 1400 (59.2) | 88 (56.1) | 0.126 |
|  | **ASIAN** | 398 (15.8) | 380 (16.1) | 18 (11.5) | **---** |
|  | **OTHER** | 270 (10.7) | 246 (10.4) | 24 (15.3) | **---** |
|  | **LATINX** | 242 (9.6) | 226 (9.6) | 16 (10.2) | **---** |
|  | **BLACK OR AFRICAN AMERICAN** | 122 (4.8) | 111 (4.7) | 11 (7.0) | **---** |
| SOCIAL SUPPORT | **SINGLE/SEPARATED** | 1079 (42.8) | 989 (41.9) | 90 (57.3) | **<0.001** |
|  | **PARTNERED** | 1441 (57.2) | 1374 (58.1) | 67 (42.7) | **---** |
| PRIMARY INSURANCE | **PPO** | 1287 (51.1) | 1214 (51.4) | 73 (46.5) | **<0.001** |
|  | **MEDICARE** | 517 (20.5) | 493 (20.9) | 24 (15.3) | **---** |
|  | **HMO/POS/EPO** | 409 (16.2) | 389 (16.5) | 20 (12.7) | **---** |
|  | **MEDICAID** | 235 (9.3) | 214 (9.1) | 21 (13.4) | **---** |
|  | **OTHER** | 72 (2.9) | 53 (2.2) | 19 (12.1) | **---** |
| *Grouped by PRIMARY INSURANCE* | | Overall  N (%) or  MEAN (SD) | REPORTED  N (%) or  MEAN (SD) | MISSING  N (%) or  MEAN (SD) | P-VALUE |
| --- | **---** | 3240 | 2363 | 877 | **---** |
| AGE AT FIRST IMAGING | **---** | 49.3 (16.8) | 49.6 (16.6) | 48.4 (17.1) | 0.075 |
| YEAR AT FIRST IMAGING | **---** | 2018.7 (3.1) | 2018.9 (3.2) | 2018.4 (3.0) | **<0.001** |
| SEX | **MALE** | 1762 (54.4) | 1269 (53.7) | 493 (56.2) | 0.217 |
|  | **FEMALE** | 1478 (45.6) | 1094 (46.3) | 384 (43.8) | **---** |
| PREFERRED LANGUAGE | **ENGLISH** | 3058 (94.4) | 2220 (93.9) | 838 (95.6) | 0.094 |
|  | **NON-ENGLISH** | 182 (5.6) | 143 (6.1) | 39 (4.4) | **---** |
| RACE/ETHNICITY | **WHITE** | 1953 (60.3) | 1400 (59.2) | 553 (63.1) | 0.168 |
|  | **ASIAN** | 508 (15.7) | 380 (16.1) | 128 (14.6) |  |
|  | **OTHER** | 341 (10.5) | 246 (10.4) | 95 (10.8) | **---** |
|  | **LATINX** | 297 (9.2) | 226 (9.6) | 71 (8.1) | **---** |
|  | **BLACK OR AFRICAN AMERICAN** | 141 (4.4) | 111 (4.7) | 30 (3.4) | **---** |
| SOCIAL SUPPORT | **SINGLE/SEPARATED** | 1365 (42.1) | 989 (41.9) | 376 (42.9) | 0.63 |
|  | **PARTNERED** | 1875 (57.9) | 1374 (58.1) | 501 (57.1) | **---** |
| SMOKING STATUS | **NEVER** | 2409 (74.4) | 1759 (74.4) | 650 (74.1) | 0.788 |
|  | **FORMER** | 646 (19.9) | 466 (19.7) | 180 (20.5) | **---** |
|  | **SMOKER** | 185 (5.7) | 138 (5.8) | 47 (5.4) | **---** |

Code Snippet 1:

SQL queries extracted patient medication prescription classes (NSAID or opioid) from the Information Commons Electronic Medical Record. Queries identified all medication classes through "medication name" fields. Additional queries identified opioid medications through the analgesic opioid "medication pharmaceutical subclass" field.

#Python code using the package duckdb

#STEP 1 - NSAID

nsaid_list = ['ACTRON','ADVIL','ALEVE','ALGIX','ANAPROX','ANSAID','ASPIRIN','ACETYLSALICYLICACID','BRUFEN','BUTAZOLIDIN','CATAFLAM','CEEOXX','CELEBREX','CELECOXIB','CEOXX','CHOLINEMAGNESIUMTRISALICYLATE','CLINORIL','CLOTAM','DAYPRO','DAYRUN','DEXDETOPROFEN','','DICLOFENAC','DIFLUNISAL','DISALCID','DOLOBID','DURAPROX','DYNASTAT','ETODOLAC','ETORICOXIB','EQUIOXX','FELDENE','FENOPROFEN','FENOPRON','FIROCOXIB','FLURBIPROFEN','','FLURWOOD','FROBEN','IBUPROFEN','INDOCIN,INDOCINSR','INDOMETHACIN','','KERAL','KETOFLAM','KETOPROFEN','KETOROLAC','LICOFELONE','LODINE,LODINEXL','LORNOXICAM','LOXOPROFEN','LOXONIN','LOXOMAC','LUMIRACOXIB','MECLOMEN','MECLOFENAMICACID','MECLOFENEMATE','MEDIPREN','MEFENAMICACID','MELOX','MELOXICAM','MESULID','MIDOL','MOBIC','MOBIFLEX','MONO-GESIC','MOTRIN','MOVALIS','NABUMETONE','NALFON','NAPRELAN','NAPROSYN','NAPROXEN','NIMALOX','NIMESULIDE','NUPRIN','NUROFEN','ORUDIS','ORUVAIL','OXAPOROZIN','OXENO','PARECOXIB','PHENYLBUTAZONE','PIROXICAM','PONSTEL','PREVICOX','PREXIGE','RAPID','RECOXA','RELAFEN','ROFECOXIB','SALFLEX','SALICYLATE','SALSALATE','SALSITAB','SPRIX','SULIDE','SULINDAC','TENOXICAM','TOLECTIN','TOLFENAMICACID','TORADOL','TRILISATEDISACLID','TUFNIL','TYLENOL','URBIFEN','VALDECOXIB(BEXTRA)','VIOXX','VOLTAREN,VOLTAREN-XR','XEFO']

nsaid_step1_query_txt = ' OR '.join(['UPPER(medicationname) LIKE \'%'+x+'%\'' for x in nsaid_list])

#STEP 2 - Weak Opioids (less addictive)

opioid_step2_list = ['TRAMADOL', 'TILIDIN', 'TILIDINE', 'CODEINE','%CODONE%','HYDROCODONE']

opioid_step2_query_txt = ' OR '.join(['UPPER(medicationname) LIKE \'%'+x+'%\'' for x in opioid_step2_list])

#STEP 3 - Strong Opioids

opioid_step3_list = ['OXYCONTIN','VICODIN','OXYCODONE','HYDROCODONE','FENTANYL','FENTANIL','%FENTAN_L%',

'MORPHINE','MORPHONE','%MORPH_NE%','MEPERIDINE','BUPRENORPHINE','DILAUDID']

opioid_step3_query_txt = ' OR '.join(['UPPER(medicationname) LIKE \'%'+x+'%\'' for x in opioid_step3_list])

medsQuery = f'''

/*

Create Date: 6/11/2024

*/

SELECT DISTINCT

*,

CASE

WHEN {opioid_step3_query_txt} THEN 'STEP 3 OPIOID'

WHEN {opioid_step2_query_txt} THEN 'STEP 2 OPIOID'

WHEN {nsaid_step1_query_txt} THEN 'STEP 1 NSAID'

ELSE 'Unspecified'

END AS medicationtype

FROM

medicationorderfact

WHERE patientdurablekey IN {tuple(patientdurablekey_workinglist)}

AND startdatekey > 0

-- for prescribed medication

AND UPPER(mode) LIKE 'OUTPATIENT'

AND

(

--FOR OPIOIDS

(

(

UPPER(medicationtherapeuticclass) LIKE 'ANALGESICS'

AND UPPER(medicationpharmaceuticalsubclass) LIKE '%OPIOID%'

AND UPPER(medicationpharmaceuticalsubclass) NOT LIKE '%NON-OPIOID%'

AND UPPER(medicationpharmaceuticalsubclass) NOT LIKE '%DIETARY SUPPLEMENT%'

AND

(

UPPER(medicationpharmaceuticalsubclass) LIKE '% AGONISTS%' --not antagonists

OR UPPER(medicationpharmaceuticalsubclass) LIKE '%CODEINE%'

OR UPPER(medicationpharmaceuticalsubclass) LIKE '%FENTANYL%'

OR UPPER(medicationpharmaceuticalsubclass) LIKE '%CODONE%'

OR UPPER(medicationpharmaceuticalsubclass) LIKE '%HYDROMORPHONE%'

OR UPPER(medicationpharmaceuticalsubclass) LIKE '%MEPERIDINE%'

OR UPPER(medicationpharmaceuticalsubclass) LIKE '%PENTAZOCINE%'

OR UPPER(medicationpharmaceuticalsubclass) LIKE '%PROPOXYPHENE%'

OR UPPER(medicationpharmaceuticalsubclass) LIKE '%TRAMADOL%'

)

)

)

OR

-- FOR NASIDs (non-steroidal anti-inflammatory drugs)

(

{nsaid_step1_query_txt}

)

OR

-- FOR Alternative Medicine

(

{alt_med_query_txt}

)

)

'''
